# Comparative microbiome analysis reveals the variation in microbial communities between ‘Kyoho’ grape and its bud mutant variety

**DOI:** 10.1371/journal.pone.0290853

**Published:** 2023-08-30

**Authors:** Tong-Lu Wei, Yu-Ping Zheng, Ze-Hang Wang, Ya-Xin Shang, Mao-Song Pei, Hai-Nan Liu, Yi-He Yu, Qiao-Fang Shi, Dong-Ming Jiang, Da-Long Guo

**Affiliations:** 1 College of Horticulture and Plant Protection, Henan University of Science and Technology, Luoyang, 471023, China; 2 Henan Engineering Technology Research Center of Quality Regulation of Horticultural Plants, Luoyang, 471023, China; 3 Library, Henan University of Science and Technology, Luoyang, 471023, China; 4 Jiangsu Red Sun Wine Industry Limited Company, Xuzhou, 221000, China; Bayero University Kano, NIGERIA

## Abstract

Microbes are an important part of the vineyard ecosystem, which significantly influence the quality of grapes. Previously, we identified a bud mutant variety (named ‘Fengzao’) from ‘Kyoho’ grapes. The variation of microbial communities in grape and its bud mutant variety has not been studied yet. So, in this study, with the samples of both ‘Fengzao’ and ‘Kyoho’, we conducted high-throughput microbiome sequencing and investigated their microbial communities in different tissues. Obvious differences were observed in the microbial communities between ‘Fengzao’ and ‘Kyoho’. The fruit and the stem are the tissues with relatively higher abundance of microbes, while the leaves contained less microbes. The fruit and the stem of ‘Kyoho’ and the stem of ‘Fengzao’ had relatively higher species diversity based on the alpha diversity analysis. *Proteobacteria*, *Enterobacteriaceae* and *Rhodobacteraceae* had significantly high abundance in ‘Fengzao’. *Firmicutes* and *Pseudomonas* were highly abundant in the stems of ‘Kyoho’, and family of *Spirochaetaceae*, *Anaplasmataceae*, *Chlorobiaceae*, and *Sphingomonadaceae*, and genera of *Spirochaeta*, *Sphingomonas*, *Chlorobaculum* and *Wolbachia* were abundant in the fruits of ‘Kyoho’. These identified microbes are main components of the microbial communities, and could be important regulators of grapevine growth and development. This study revealed the differences in the microbial compositions between ‘Kyoho’ and its bud mutant, and these identified microbes will be significant resources for the future researches on the quality regulation and disease control of grapevines.

## Introduction

Grape is an important cash crop, and China is one of the most important grape-growing countries with the production and area of table grapes ranked top around the world for a long time [1]. Microbes are an important part of the vineyard ecosystem, which participate in multiple physiological and biochemical processes during the grapevine cultivation [2,3]. In grapevine agriculture, disease occurs frequently, resulting in greatly-reduced production and huge economic losses. Besides, during postharvest storage of grapes, many endophytic bacteria also easily cause rot and deterioration, affecting normal sales. However, many grape growers and sellers are mostly unaware of the microbes that cause these diseases. So, it is of great significance to study the microbiome composition and microbial diversity in grapevines [4].

The microbial compositions are various for different tissues of the grapevines. In the rhizosphere (the area around the root), microbes are more numerous and complex due to their direct contact with the soil [5–7]. Some important microbes have been identified from the grapevine rhizosphere, such as *Clostridium*, *Bacillus*, *Rhizobium*, *Acinetobacter*, *Streptococcus*, *Paenibacillus* and other bacteria, as well as some fungi, such as *Filobasidium capsuligenum*, *Aureobasidium pullulans* and *Hanseniaspora* [4,8–10]. Rhizosphere microbes are affected by plant uptake, root exudates, and soil activities. At the same time, rhizosphere microbes also directly affect nutrient uptake, nutrient utilization, growth, development, and disease occurrence for the grapevines [11–13]. For example, *Proteobacteria*, with high abundance in grapevines, is involved in the cycling process of major nutrient elements, which can improve nitrogen utilization efficiency [14,15]. Microbial diversity in the phyllosphere (or leaf surface) is also one of the focuses of current researches. Leaves are the main dynamic habitats for microbes. Phyllospheric microbes mainly affect the fixation of carbon and nitrogen, thereby affecting plant growth and development [3,7]. In addition, some harmful microbes in the phyllosphere are also the main sources of some diseases [6,16,17]. Grape berries are also important habitats for microbes, which directly affect the economic value and nutritional value of grapes, especially for wine grapes, and the microbes inhabiting wine grapes have a direct impact on the aroma, color and quality of wine [18,19]. The microbes on grape berries can also cause some serious diseases, resulting in a decrease in yield and quality. For example, *Alternaria* sp., a bacterium on grape berries, can produce a variety of toxic metabolites, which cause the disease of black spot, and even giving rise to the poisoning and cancer after human ingestion [20,21]. Beyond rhizosphere, phyllosphere and berry, some studies have focused on the microbes in the other tissues of the grapevines, like *Xanthobcter*, *Xanthomonas*, *Cellulomonas*, and *Xylella* from the stems, and *Pseudomonas* sp. and *Bacillus* ssp. from the flowers [13,15].

The diversity of microbial community has been the focus for the researchers in microbiology, ecology and phytopathology in recent years [5,22]. By studying the dynamic changes of microbial community, we can understand the ecological functions of microbes and optimize community structure, contributing to the control and prevention of plant diseases. The current research approaches on microbial diversity has extended from traditional microorganism culture to high-throughput sequencing methods [23]. It has been very easy to understand all the microbial species and compositions of plants through high-throughput sequencing, which is commonly referred to as microbiome [24,25]. Through the comparative study of microbiome, we can systematically analyze the effects of different varieties, different ecological environments, and different treatment factors on the microbial community of fruit trees, so as to better guide the production and disease control for the orchard.

The microbiome diversity has been explored in grapevines not only in various plant parts (like berry, leaf, root, bark and bud), but also in various grape varieties. Awad et al. [26] investigated the microbiome in the tissues of bud and bark in 37 different grapevine varieties under the same viticulture environment, revealing that the genotypes of grapevines may also influence microbiome diversity regardless of growth conditions. At the same time, Awad et al. [27] further confirmed this conclusion by exploring microbial diversity in 36 grapevine varieties, indicating the impact of the genotypes and the phenological stages on the microbial communities. These researches unveiled and characterized the microbiome diversity in grapevines, suggesting that it is necessary for researchers to identify more microbial species in more tissues and terroirs. Except for these two studies, the researches on grapevine’s microbial communities are inadequate. On the other hand, current studies on microbial diversity mainly focus on different cultivars or species [14,15,28], while some natural mutant varieties (such as bud mutant in many horticultural plants) have not been studied yet.

Previously, we identified a bud mutant from the ‘Kyoho’ grape, named as ‘Fengzao’, which is typically characterized by early-ripening, with a maturity period of 30 days earlier than ‘Kyoho’ [29]. We have also compared the developmental process, the fruit physiology, and the transcriptome between the two cultivars [30–32], but the microbiome differences between them have not been investigated. Besides, the variation of microbial community for bud mutant variety in horticultural plants has not been studied yet. Therefore, in this study, we systematically compared the microbiome in different tissues of ‘Kyoho’ and ‘Fengzao’ in order to dissect the microbiome diversity and the microbial composition structure in both grapevine and its bud mutant variety, which will provide an important reference for relevant researches and lay a foundation for the future studies on the mechanisms underlying the microbial community’ variation.

## Materials and methods

### Plant materials

Grapevines of ‘Kyoho’ and ‘Fengzao’ were planted in the experimental fields of Henan University of Science and Technology (Luoyang, China) under the same viticulture management practices. Samples of fruit, stem and leaf were collected on April 15, 2020. Three trees were selected for ‘Fengzao’ and ‘Kyoho’, and a bunch of berries, 5 leaves, and 10 cm-length stem segments were respectively taken from each vine. Pooled samples of the stems, leaves and berries from each variety were immediately frozen in liquid nitrogen, and stored in a -80°C for further use. The samples of fruit (F), leaf (L) and stem (S) labeled as ‘KF’, ‘KL’, ‘KS’ for ‘Kyoho’ variety and as ‘FF’, ‘FL’, ‘FS’ for ‘Fengzao’ variety were formed, respectively (Table 1).

**Table 1:** Experimental samples in this study.

Variety	Tissue	Abbreviation
‘Kyoho’	Fruit	KF
	Stem	KS
	Leaf	KL
‘Fengzao’	Fruit	FF
	Stem	FS
	Leaf	FL

### Library construction and sequencing

Total DNA were extracted from the stored 6 samples using the DNeasy^®^ PowerFood^®^ Microbial Kit (MoBio Laboratories Inc., CA, USA) according to the manufacturer’s instruction, and the entire samples were used so that both epiphytic or endophytic microbial DNA were obtained. The DNA was quantified with Qubit 4.0 fluorometer (Invitrogen, CA, USA), and the DNA quality was checked using a NanoDrop spectrophotometer (Thermo Scientific, CA, USA). Primers were determined according to the conservative regions of microbiome, and the 16S rRNA (V3+V4 region) gene and internal transcribed spacer 1 (ITS1) loci was used as previously described by Kamilari et al. [33]. Primer sequences for 16S rRNA are: V3: 5’ -TCGTCGGCAGCGTCAGATGTGTATAAGAGACAG-3’ and V4: 5’-GTCTCGTGGGCTCGGAGATGTGTATAAGAGACAG-3’; for ITS1: 5’-GAGATCCRTTGYTRAAAGTT-3’ and 5’-NNNNNNNNCTACCTGCGGARGGATCA-3’. After connecting with the adaptor, PCR amplification was performed. The products were purified, quantified and homogenized to form sequencing libraries. The constructed libraries were subjected to library-quality inspection, and the qualified libraries were sequenced by Illumina HiSeq 2500 platform. Raw image data files obtained by high-throughput sequencing are converted into raw sequenced reads by base calling analysis. The results are stored in FASTQ file format, which contained the detailed sequence information of reads and their corresponding sequencing quality information. The generated data are available in the NCBI SRA repository under the BioProject ID: PRJNA939915 (accession numbers: SRX19531416-SRX19531421).

### Data preprocessing

According to the overlaps of the reads, the paired-end sequence data obtained by Hiseq were merged into sequence tags, which were filtered by quality control according to the following three steps: (1) paired-end reads were spliced using FLASH v1.2.7 software (http://ccb.jhu.edu/software/FLASH/) based on a criterion: minimum overlapping length is 10 bp and maximum mismatching ratio of overlapping regions is 0.2, to obtain the raw tags data. (2) The raw tags data was filtered using Trimmomatic v0.33 software (http://www.usadellab.org/cms/?page=trimmomatic) with the parameter set as a window of 50 bp. If the average quality value in the window was lower than 20, the base at the back end would be cut from the window, and the tags with length less than 75% would be eliminated. After this, the high-quality tags data (clean tags) were obtained. (3) Using UCHIME v4.2 software (http://drive5.com/usearch/manual/uchime_algo.html), the chimeric sequences were identified and removed to obtain the final effective clean tags.

### OTU (operational taxonomic unit) analysis

All obtained tags were divided into different OTUs. Generally, if the similarity between sequences is higher than 97%, it can be defined as an OTU, and each OTU corresponds to a representative sequence. Each OTUs were obtained with the UCLUST in QIIME (version 1.8.0) software [34] at a 97% similarity level.

### Species annotation and taxonomic analysis

The representative sequences of OTUs were aligned with the microbial reference database (Silva, Release 119, http://www.arb-silva.de; UNITE, Release 7.0, http://unite.ut.ee/index.php) to obtain the classification information, and then the community composition of each sample was counted at each level (phylum, class, order, family, genus, species). QIIME (version 1.8.0) software [34] was used to generate figures showing species abundance at different taxonomic levels, and R package tools were used to draw maps showing the bacterial community structure at each taxonomic level.

### Alpha diversity analysis

Mothur (version v.1.30) software (http://mothur.org/) was used to conduct the Alpha diversity analysis. To compare diversity between samples, the number of sequences contained in the samples was normalized during analysis. The Alpha diversity was analyzed with four indicators, including Chao1, Ace, Shannon, and Simpson. The Rarefaction Curve and the Shannon Index Curve were drawn with Mothur software and R package to To verify whether the amount of sequencing data is sufficient to reflect the species diversity in the samples.

### Beta diversity analysis

QIIME (version 1.8.0) software [34] was used for beta diversity analysis to compare the differences in species diversity among different samples. Based on the results of Beta diversity analysis, PCoA (Principal Coordinates Analysis) [35] and NMBS (Non-metric Multi-Dimensional Scaling) [36] maps were drawn respectively using R package tools.

### Analysis of 16S functional genes

PICRUSt2 (v2.4.0) software [37] (https://github.com/picrust/picrust2) was used to infer the functional gene composition in the samples through 16S sequencing, so as to analyze the functional differences between different samples or groups. Firstly, the generated OTUs were standardized, as different genus or species have different 16S copy numbers. Then, through the greengene id corresponding to each OTU, the COG (Clusters of Orthologous Groups of proteins) family information of each OTU can be obtained. The COG abundance and the abundance of each functional category could be calculated by obtaining the KO, Pathway, EC information from the COG database (https://www.ncbi.nlm.nih.gov/COG/). At the genus level, pairwise tests for significant differences between different samples were performed with two-sample T-TEST method in STAMP software (https://beikolab.cs.dal.ca/software/STAMP) (for significant level set as P-value < 0.05).

## Results

### Sequencing statistics

To understand the microbial community of grapevines, we conducted high-throughput microbiome sequencing with samples of different tissues of ‘Kyoho’ and ‘Fengzao’. A total of 1,390,484 pairs of Reads were sequenced from the 6 samples, and 1,154,430 clean tags were generated after splicing and filtering, with an average of 192,405 clean tags generated per sample. The data quality was evaluated by statistical data processing, mainly by statistics of sequence number, sequence length, GC content, Q20 and Q30 quality value, effective value and other parameters in each sample (S1Table in S1 File). After quality control, the data was used for subsequent analysis. The length distribution of obtained clean tags was counted in the corresponding length range of each sample, and the widest distribution of clean tags length is 440 to 450 nt (nucleotide) for all samples (S1 Fig in S1 File).

### OTU (operational taxonomic unit) analysis

**Fig 1:**
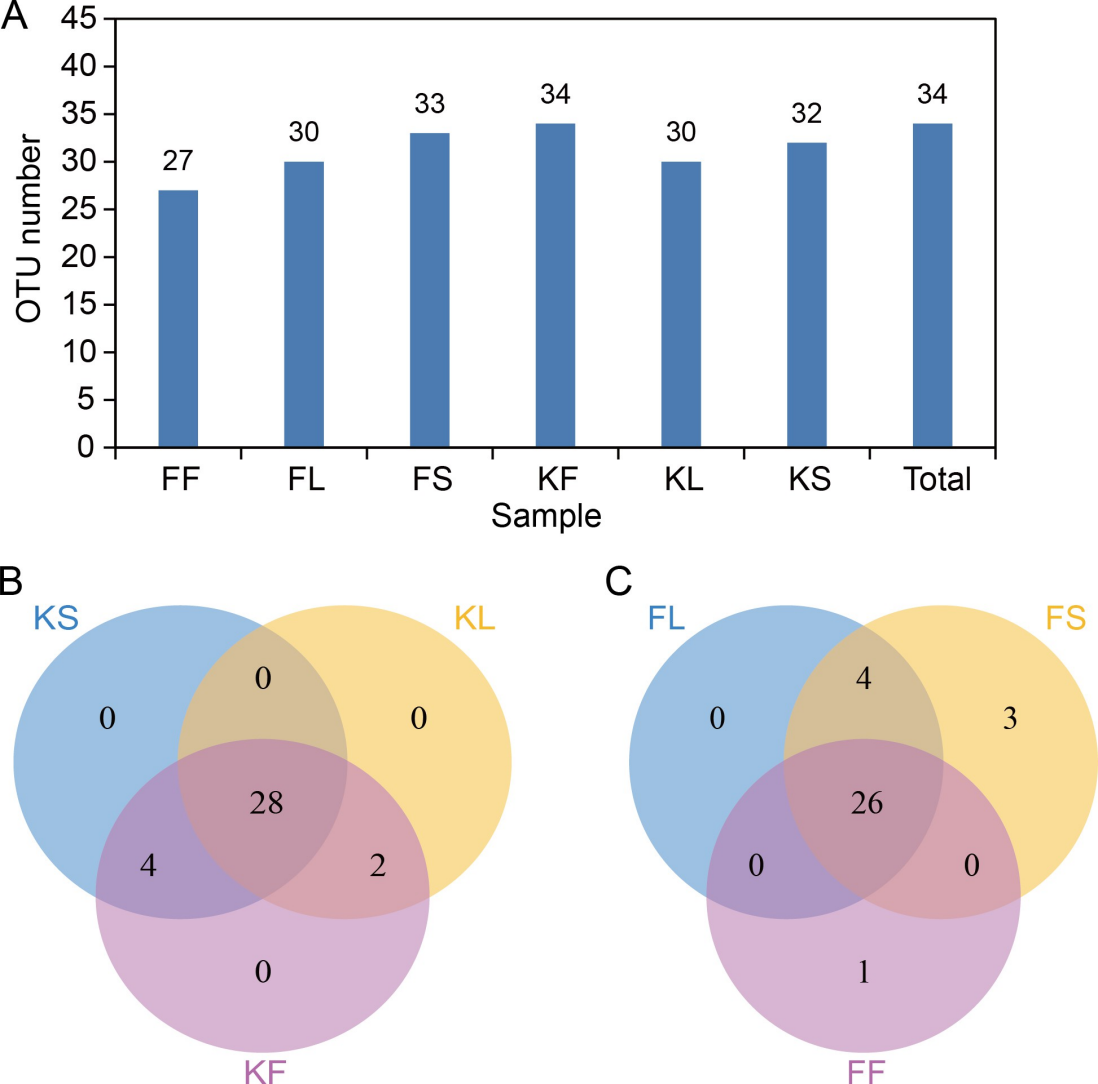
Statistics of OTU (operational taxonomic unit) in different samples. (A) OTU number in each sample and total number of the identified OTU. (B-C) Venn diagrams showing the OTU numbers among different samples of ‘Kyoho’ (B) and ‘Fengzao’ (C). ‘FF’, ‘FL’ and ‘FS’ indicate samples of fruit, leaf and stem in ‘Fengzao’; ‘KF’, ‘KL’ and ‘KS’ indicate samples of fruit, leaf and stem in ‘Kyoho’.

OTU is artificially-assigned taxon (strain, species, genus, group, etc.) for the convenience of analysis in phylogenetic studies or population genetics studies [20]. In general, if the similarity between sequences is higher than 97%, it can be defined as an OTU, and each OTU corresponds to a representative sequence [38]. Accordingly, at 97% similarity level, we conducted OTU analysis with the obtained sequences, and performed taxonomic annotation on OTU based on Silva (Release 119, bacteria) and UNITE (Release 7.0, fungi) taxonomic databases. A total of 34 OTUs were obtained from the six samples (Table 2, S2 Table in S1 File, Fig 1A), with 27 OTUs in FF, 30 OTUs in FL, 33 OTUs in FS, 34 OTUs in KF, 30 OTUs in KL, and 32 OTUs in KS (Fig 1A). The Venn diagrams showed that 28 OTUs were commonly present in different tissues of ‘Kyoho’ (KF, KL, KS), and 26 OTUs were commonly present in different tissues of ‘Fengzao’ (FF, FL, FS). Only a few OTUs are tissue specific (6 in ‘Kyoho’ and 8 in ‘Fengzao’) (Fig 1B and 1C).

**Table 2:** Information of the 34 OUTs from each sample.

OTU ID	Sequence count						Taxonomy
	FF	FL	FS	KF	KL	KS	
OTU477	0	28	5	47	29	5	k__Bacteria; p__Spirochaetae; c__Spirochaetes; o__Spirochaetales; f__Spirochaetaceae; g__Spirochaeta
OTU623	18	20	16	21	18	6	k__Bacteria; p__Cyanobacteria; c__Chloroplast
OTU893	15	14	12	8	9	4	k__Bacteria; p__Cyanobacteria; c__Chloroplast; o__uncultured_bacterium;
OTU1418	120	29	59	77	26	27	k__Bacteria; p__Cyanobacteria; c__Chloroplast; o__Gerbera_hybrid_cultivar;
OTU1664	291	162	245	238	94	148	k__Bacteria; p__Proteobacteria; c__Alphaproteobacteria; o__Rickettsiales; f__mitochondria
OTU2296	0	4	32	31	2	6	k__Bacteria; p__Spirochaetae; c__Spirochaetes; o__Spirochaetales; f__Spirochaetaceae; g__uncultured; s__uncultured_bacterium
OTU2554	48	36	59	36	33	32	k__Bacteria; p__Cyanobacteria; c__Chloroplast; o__uncultured_bacterium;
OTU2622	92	50	13	85	14	4	k__Bacteria; p__Proteobacteria; c__Alphaproteobacteria; o__Rhizobiales
OTU2892	10	10	15	12	12	6	k__Bacteria; p__Cyanobacteria; c__Chloroplast
OTU2939	19	21	15	14	11	16	k__Bacteria; p__Cyanobacteria; c__Chloroplast
OTU3079	35	2	7	17	6	1	k__Bacteria; p__Proteobacteria; c__Alphaproteobacteria; o__Rhodobacterales; f__Rhodobacteraceae
OTU3218	32	15	20	19	19	19	k__Bacteria; p__Cyanobacteria; c__Chloroplast
OTU3488	4	7	7	295	22	37	k__Bacteria; p__Proteobacteria; c__Alphaproteobacteria; o__Rickettsiales; f__Anaplasmataceae; g__Wolbachia
OTU3613	21	20	7	11	12	11	k__Bacteria; p__Cyanobacteria; c__Chloroplast
OTU3748	0	0	20	130	5	0	k__Bacteria; p__Chlorobi; c__Chlorobia; o__Chlorobiales; f__Chlorobiaceae; g__Chlorobaculum
OTU4063	9	11	16	13	8	11	k__Bacteria; p__Cyanobacteria; c__Chloroplast
OTU4313	359	0	0	287	0	441	k__Bacteria; p__Firmicutes; c__Bacilli; o__Lactobacillales; f__Enterococcaceae; g__Tetragenococcus
OTU4701	68	22	14	81	6	8	k__Bacteria; p__Proteobacteria; c__Alphaproteobacteria; o__Sphingomonadales; f__Sphingomonadaceae; g__Sphingomonas; s__uncultured_Sphingomonas_sp.
OTU4736	36	32	43	53	33	20	k__Bacteria; p__Proteobacteria; c__Alphaproteobacteria; o__Rickettsiales; f__mitochondria
OTU5662	30	24	19	25	27	16	k__Bacteria; p__Cyanobacteria; c__Chloroplast
OTU5889	0	0	22	1	0	50	k__Bacteria; p__Synergistetes; c__Synergistia; o__Synergistales; f__Synergistaceae; g__uncultured; s__uncultured_bacterium
OTU6041	13	10	11	14	7	8	k__Bacteria; p__Cyanobacteria; c__Chloroplast
OTU6515	49	49	35	40	24	25	k__Bacteria; p__Proteobacteria; c__Alphaproteobacteria; o__Rickettsiales; f__mitochondria
OTU6717	85	70	86	115	62	65	k__Bacteria; p__Cyanobacteria; c__Chloroplast; o__Gerbera_hybrid_cultivar;
OTU6854	0	44	47	6	0	1	k__Bacteria; p__Firmicutes; c__Clostridia; o__Clostridiales; f__Ruminococcaceae; g__Incertae_Sedis; s__uncultured_bacterium
OTU6916	16	15	12	16	11	5	k__Bacteria; p__Cyanobacteria; c__Chloroplast; o__Gerbera_hybrid_cultivar;
OTU6922	54	2	1	42	41	24	k__Bacteria; p__Cyanobacteria; c__Chloroplast; o__uncultured_bacterium;
OTU7563	0	0	15	20	0	30	k__Bacteria; p__Proteobacteria; c__Gammaproteobacteria; o__Pseudomonadales; f__Pseudomonadaceae; g__Pseudomonas
OTU7879	11	27	285	14	4	0	k__Bacteria; p__Proteobacteria; c__Gammaproteobacteria; o__Enterobacteriales; f__Enterobacteriaceae; g__Enterobacter
OTU7914	30425	11094	20369	23534	6629	7682	k__Bacteria; p__Proteobacteria; c__Alphaproteobacteria; o__Rickettsiales; f__mitochondria
OTU8110	0	1	68	9	3	2	k__Bacteria; p__Proteobacteria; c__Betaproteobacteria; o__Burkholderiales; f__Oxalobacteraceae
OTU8121	203992	193188	163517	191117	142317	121669	k__Bacteria; p__Cyanobacteria; c__Chloroplast
OTU8408	25	29	18	26	12	14	k__Bacteria; p__Cyanobacteria; c__Chloroplast
OTU8592	14	15	9	11	9	6	k__Bacteria; p__Cyanobacteria; c__Chloroplast

### Species annotation and taxonomic analysis

In order to analyze the community composition of each sample, we compared the representative sequence of OTU with the microbial reference database to obtain the corresponding species classification information for each OTU, and then obtained the classification information of each OTU at various levels (phylum, class, order, family, genus) (Table 2). By statistics, at the level of phylum, the microbial community with a relatively higher abundance in the six samples were *Cyanobacteria* and *Proteobacteria* (S2A Fig in S1 File). At the level of class, *Chloroplast* and *Alphaproteobacteria* had higher abundance in the six samples (S2B Fig in S1 File). And at the level of order and family, *Rickettsiales* and *mitochondria* with known functions had relatively higher abundance in the six samples, while much more microbial communities with highest abundance were unknown (S2C and S2D Fig in S1 File). At the level of genus, eight OTUs had annotation, like OTU477 (*Spirochaeta*), OTU3488 (*Wolbachia*), OTU3748 (*Chlorobaculum*), OTU4313 (*Tetragenococcus*), OTU4701 (*Sphingomonas*), OTU6854 (*Incertae_Sedis*), OTU7563 (*Pseudomonas*), and OTU7879 (*Enterobacter*) (Table 2).

**Fig 2:**
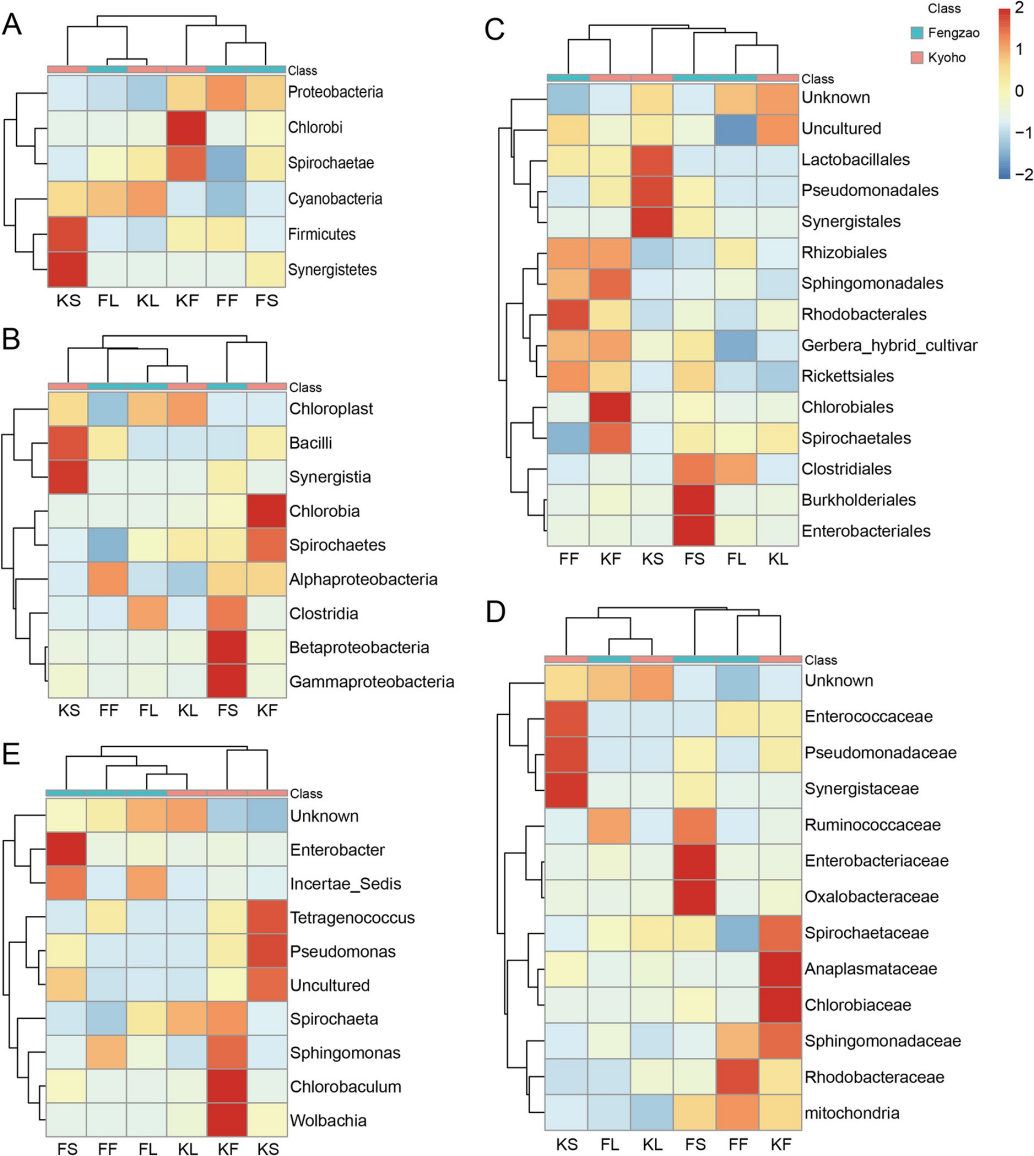
Clustering heat maps of bacterial abundance at all levels, including phylum (A), class (B), order (C), family (D), genus (E). Horizontal clustering refers to sample information and vertical clustering refers to bacterial information. Heatmap shows the bacterial abundance in each sample, with red and blue color representing high and low abundance, respectively.

To specify and compare the bacterial compositions in the six samples, we used heatmaps to show the relative abundance in each sample at different levels (Fig 2). At the level of phylum, *Proteobacteria* had the highest abundance in the sample of FF; *Chlorobi* and *Spirochaetae* had the highest abundance in KF; *Cyanobacteria* had the highest abundance in KL; *Firmicutes* and *Synergistetes* had the highest abundance in KS (Fig 2A). At the level of class, *Chloroplast* and *Alphaproteobacteria* had the highest abundance in KL and FF, respectively; *Bacilli* and *Synergistia* had the highest abundance in KS; *Chlorobia* and *Spirochaetes* had the highest abundance in KF; *Clostridia*, *Betaproteobacteria* and *Gammaproteobacteria* had the highest abundance in FS (Fig 2B). At the level of order, *Lactobacillales*, *Pseudomonadales* and *Synergistales* had the highest abundance in KS; *Rhizobiales*, *Sphingomonadales*, *Rhodobacterales*, *Gerbera_hybrid_cultivar* and *Rickettsiales* had relatively higher abundance in FF and KF, indicating their abundance in fruit samples; *Chlorobiales* and *Spirochaetales* had the highest abundance in KF; *Clostridiales*, *Burkholderiales* and *Enterobacteriales* had the highest abundance in FS (Fig 2C). At the level of family, the microbial communities with the highest abundance were *Enterococcaceae*, *Pseudomonadaceae*, and *Synergistaceae* in the sample of KS, *Ruminococcaceae*, *Enterobacteriaceae*, and *Oxalobacteraceae* in FS, *Spirochaetaceae*, *Anaplasmataceae*, *Chlorobiaceae*, and *Sphingomonadaceae* in KF, *Rhodobacteraceae* and *mitochondria* in FF (Fig 2D). At the level of genus, *Enterobacter* and *Incertae_Sedis* had the highest abundance in FS; *Tetragenococcus* and *Pseudomonas* had the highest abundance in KS; *Spirochaeta*, *Sphingomonas*, *Chlorobaculum* and *Wolbachia* had the highest abundance in KF (Fig 2E).

**Fig 3:**
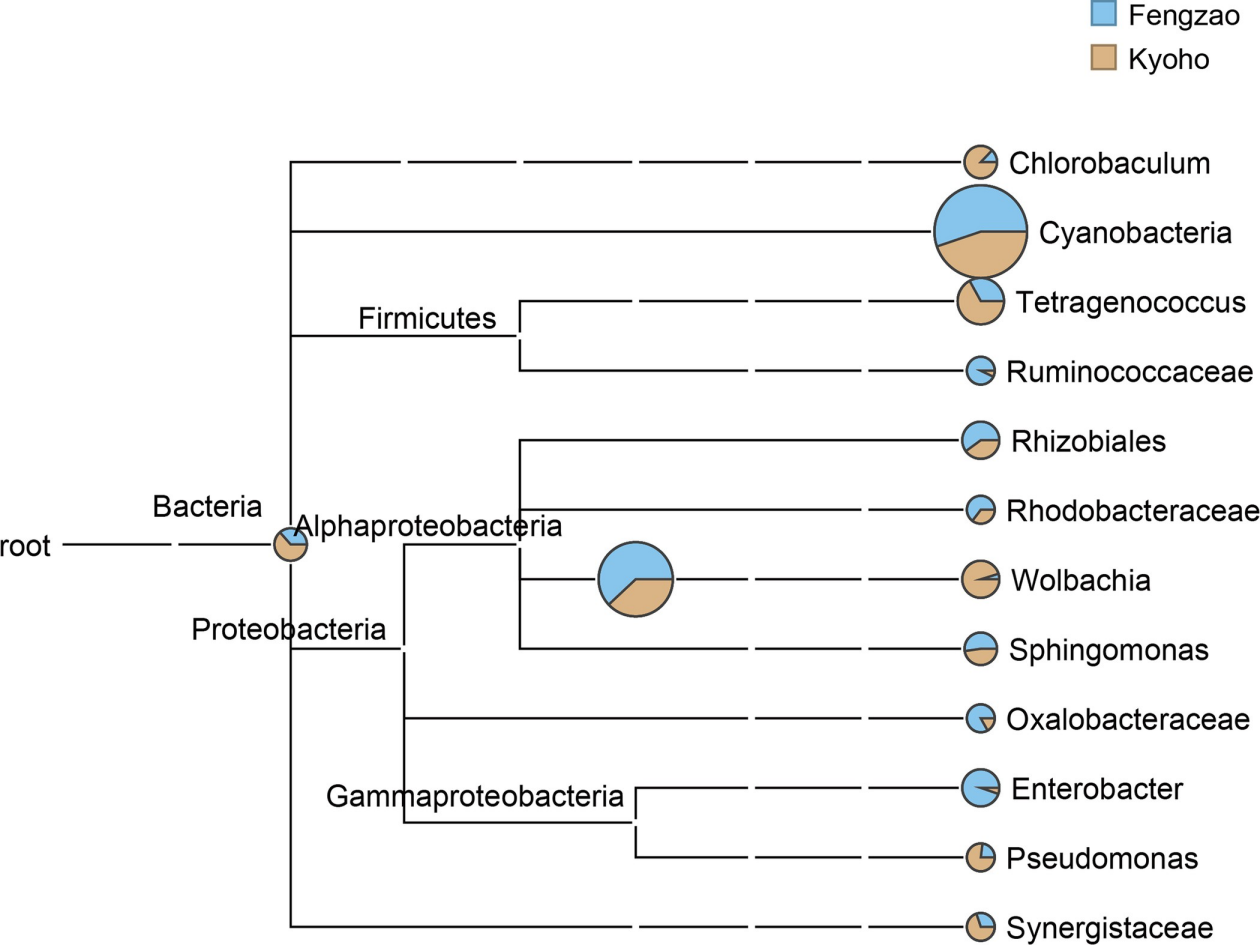
Tree diagram showing the bacterial abundance at each taxonomic level in ‘Fengzao’ and ‘Kyoho’. The bacterial abundance is compared between ‘Fengzao’ (blue) and ‘Kyoho’ (gray) with pie charts.

Additionally, we also returned our sequenced OTU information to the taxonomic system of NCBI database, so as to comprehensively understand the evolutionary relationships and abundance differences of all microbes from the samples. The evolutionary tree showed the relationship of all microbial communities and the differences of their relative abundance between the samples of ‘Kyoho’ and ‘Fengzao’ (Fig 3). The results indicated obvious abundance differences between ‘Kyoho’ and ‘Fengzao’. For example, *Cyanobacteria*, with highest abundance among all microbial communities, had higher bacterial abundance in ‘Fengzao’ than ‘Kyoho’; *Ruminococcaceae*, *Rhizobiales*, *Rhodobacteraceae*, *Oxalobacteraceae*, and *Enterobacter* all had relatively higher abundance in ‘Fengzao’ than ‘Kyoho’; while *Chlorobaculum*, *Tetragenococcus*, *Wolbachia*, *Pseudomonas*, and *Synergistaceae* had relatively higher abundance in ‘Kyoho’ than ‘Fengzao’ (Fig 3).

### Alpha diversity analysis

Alpha diversity reflects the species diversity within a single sample, which can be measured by multiple indexes, like Ace, Chao1, Simpson and Shannon [39]. A larger value of Chao1, Ace and Shannon, and a smaller value of Simpson indicate a higher species diversity of the sample. So, based on Ace and Chao1, the species diversity ranking for the six samples is KF, FS, KS, FL, KL, and FF. And based on Simpson and Shannon, this ranking is FF, KF, FS, KS, FL, and KL (Table 3). We also calculated the Coverage of the sample library, which indicated whether the sequencing results represent the true situation of the microbes in the sample, with a higher value representing a higher probability that all sequences in the sample has been measured. The results showed that the Coverage values of the six samples were all very high (more than 0.9999) (Table 3). In addition, we also plotted the Rarefaction curve and the Shannon Index curve to verify whether the amount of sequencing data was sufficient to reflect the species diversity in the samples (S3Fig in S1 File). The two kinds of curves both tended to be flat for all the six samples, indicating that the sequencing data was reasonable and saturated, and no additional sequencing was needed.

**Table 3:** Alpha diversity index statistics.

Sample_ID	OTU	Ace	Chao1	Simpson	Shannon	Coverage
FF	27	27	27	0.76447	0.437607	1
FL	30	30.570286	30	0.890567	0.245818	0.999995
FS	33	33.24112	33	0.792343	0.403951	0.999995
KF	34	34.377086	34	0.791338	0.414919	0.999995
KL	30	30	30	0.908121	0.217048	1
KS	32	32.554171	32.5	0.874068	0.288689	0.999985

(Sample_ID: The sample name; OTU: The number of OTUs; Ace, Chao1, Simpson and Shannon represent each index respectively; Coverage indicates the coverage of the sample library.)

### Beta diversity analysis

**Fig 4:**
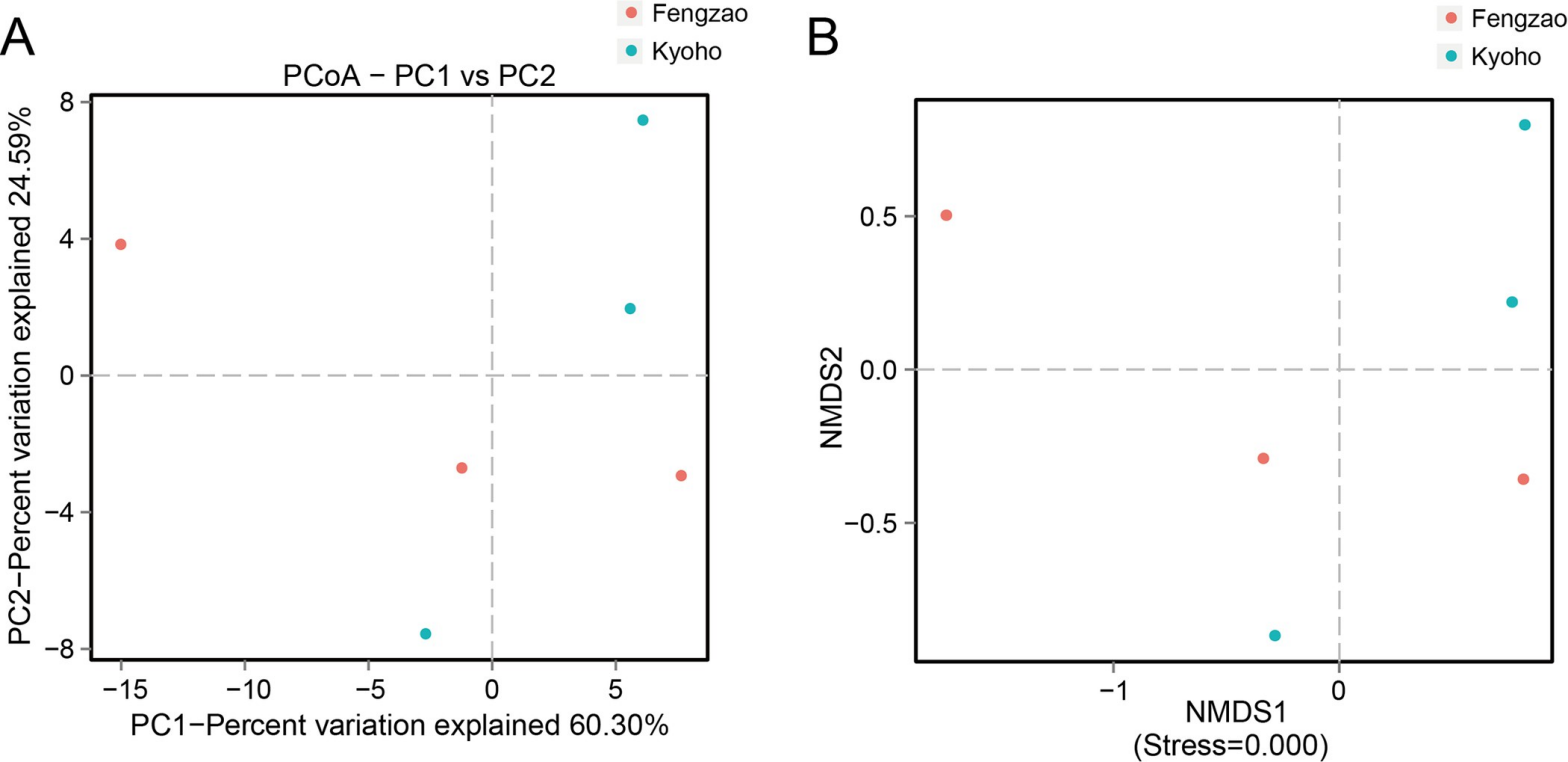
PCoA (principal coordinates analysis) (A) and NMDS (non-metric multi-dimensional scaling) diagrams (B) showing sample diversity in ‘Fengzao’ and ‘Kyoho’. (A) The red and blue dots represent samples of ‘Fengzao’ and ‘Kyoho’, respectively. Horizontal and vertical coordinates are the two characteristic values that lead to the biggest difference between samples, and the influence degree is reflected by the percentage. (B) The red and blue dots represent samples of ‘Fengzao’ and ‘Kyoho’, respectively. When the Stress is less than 0.2, it indicates that the NMDS analysis has reliability.

We employed two methods, PCoA (Principal coordinates analysis) and NMDS (Non-metric multi-dimensional scaling), to conduct beta diversity analysis, in order to further investigate the differences of microbial communities among the six samples. PCoA and NMDS exhibited similar results, both showing that samples of ‘Kyoho’ and ‘Fengzao’ were roughly divided into two classes (Fig 4), indicating the diversity of microbial communities between ‘Kyoho’ and ‘Fengzao’.

### Prediction of the functional potential of the microbial community

**Fig 5:**
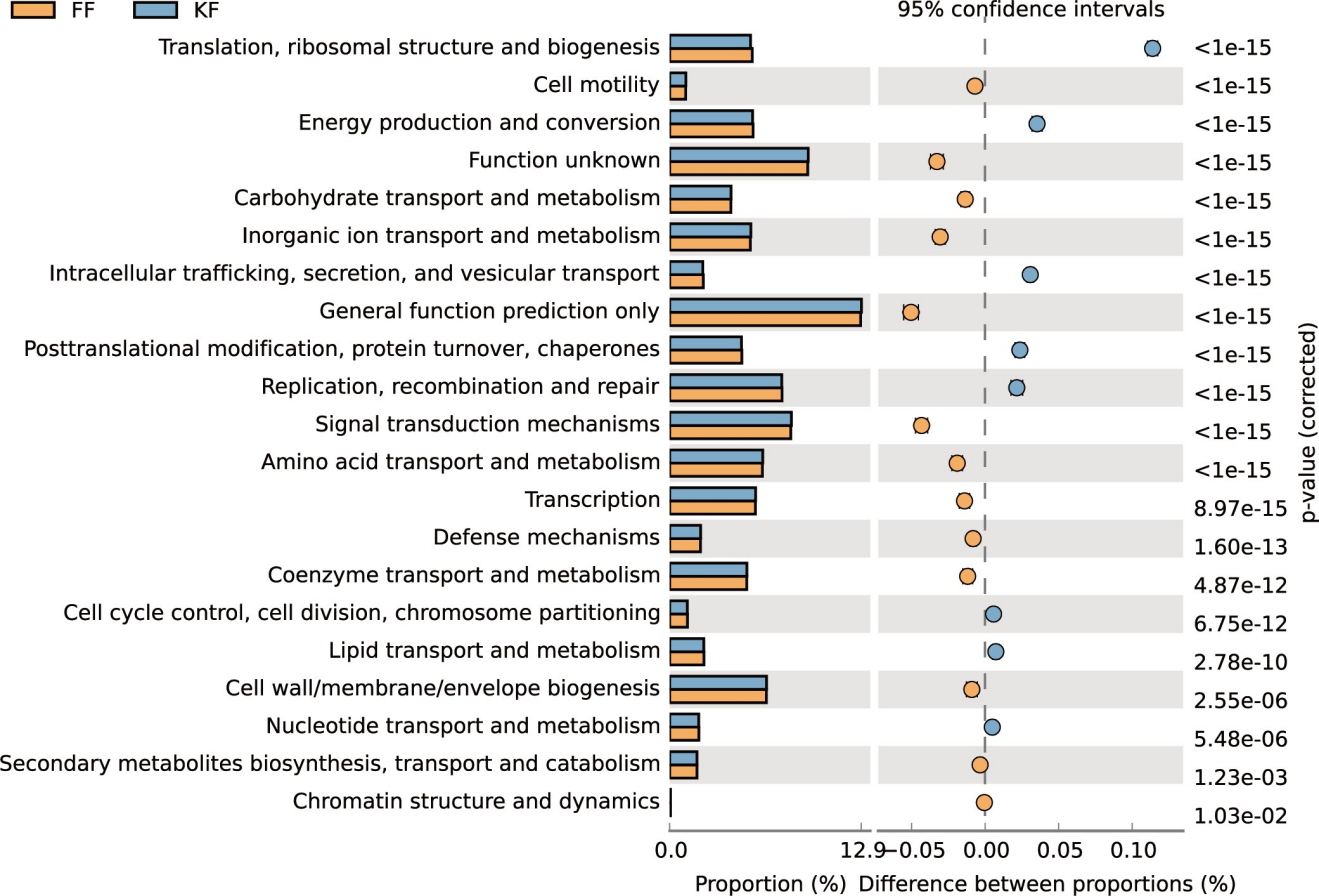
Analysis of COG metabolic pathways in fruits between ‘Fengzao’ (FF) and ‘Kyoho’ (KF). The figure shows the abundance ratio of different functions in the two cultivars. The middle bar plots and dot plots shows the difference ratio of different functions under the 95% confidence intervals, and the values on the right show the p values.

In order to predict the functional potential of the microbial communities in different samples, we mapped the obtained OTUs to the corresponding COG (Clusters of orthologous groups of proteins) database to calculate the abundance of each COG, and performed pairwise tests for significant differences between different samples at the genus level. The different tissues (fruit, leaf and stem) of ‘Kyoho’ and ‘Fengzao’ were analyzed, respectively. The predicted COG pathway with most obviously differences between ‘Kyoho’ and ‘Fengzao’ was ‘Translation, ribosomal structure and biogenesis’ (Fig 5, S4-S5 Fig in S1 File). The results from fruit, leaf and stem were similar, with some predicted pathways identified in all the three tissues, like ‘Translation, ribosomal structure and biogenesis’, ‘Cell motility’, ‘Energy production and conversion’, ‘Function unknown’, ‘Carbohydrate transport and metabolism’, ‘Inorgenic ion transport and metabolism’, ‘Intracellular trafficking, secretion, and vesicular transport’, ‘General function prediction only’, and so on (Fig 5).

## Discussion

Microbes are an important part of the grape ecosystem, which directly affect the yield, quality, stress resistance, growth and development of grapes. Especially for wine grapes, the microbial composition and content directly determine the quality of wine product [40]. ‘Kyoho’ is mainly used as table grapes, but recent studies have also shown that the microbes inside table grapes can also significantly affect grape quality and disease resistance [41]. However, studies on the microbial composition of table grapes are far behind that of wine grapes, and most of them focused on the rhizosphere, with relatively less attention paid to leaves, fruits, and stems [26,27,42,43]. Therefore, in this study, we investigated the microbiome in leaves, stems and fruits of ‘Kyoho’, a representative of table grapes. Most importantly, we previously identified a bud mutant derived from ‘Kyoho’, namely ‘Fengzao’ [29], and conducted deep studies on the comparisons of maturity period, fruit quality, physiological and molecular mechanisms of fruit development between ‘Fengzao’ and ‘Kyoho’ [29–31]. Considering the poor knowledge of microbial variation between plants and their bud mutants, especially for horticultural plants, this bud mutant germplasm can be a good resource for the further studies. Therefore, in this study, we investigated the microbial community’s variation with ‘Kyoho’ and ‘Fengzao’ using high-throughput sequencing, and the results assessed the microbial communities’ structure and diversity in various plant tissues in two grape varieties.

We identified 34 OTUs from the different grape tissues of ‘Kyoho’ and ‘Fengzao’ (Fig 1A). Although the identified microbial communities are not so rich, many representative microbes (such as *Proteobacteria*, *Firmicutes*, *Synergistetes*, *Pseudomonadales*, etc.) were identified, which are common in the horticultural plants as revealed by the previous studies [44–46]. The microbes we identified were mainly endogenous microbes (or named endophyte) which existed in the interior of plants. The communities of endophytes and epiphytes are usually different, as the epiphytes directly contact with the external environment and were influenced by the growth conditions, while the endophytes are relatively more stable with less microbial diversity in a same plant individual. So, the endophytes of plants may be less abundant than the epiphytes on the surface of the plant bodies (especially the rhizosphere) [22], while to some extent, the endophytes might affect plant normal growth even more, for example many diseases are caused by the endophytes. Previous studies mainly focused on the epiphyte [47], but in fact, the endophyte might play a more significant role for plants [25]. In recent years, more and more studies have started to investigate the endogenous microbes of plants, including *Arabidopsis* [25,28,48], apples [49] and grapes [19]. Lundberg et al. [25] rigorously defined the rhizosphere and the endophytic compartment (within the root) in *Arabidopsis*, and revealed the important functions of endophytic microbiome for plant-microbe interactions. So, while continuing to pay attention to rhizosphere microbes, we should also strengthen the researches on the endogenous microbes of other plant tissues (like leaf, fruit, stem, flower, etc.).

**Fig 6:**
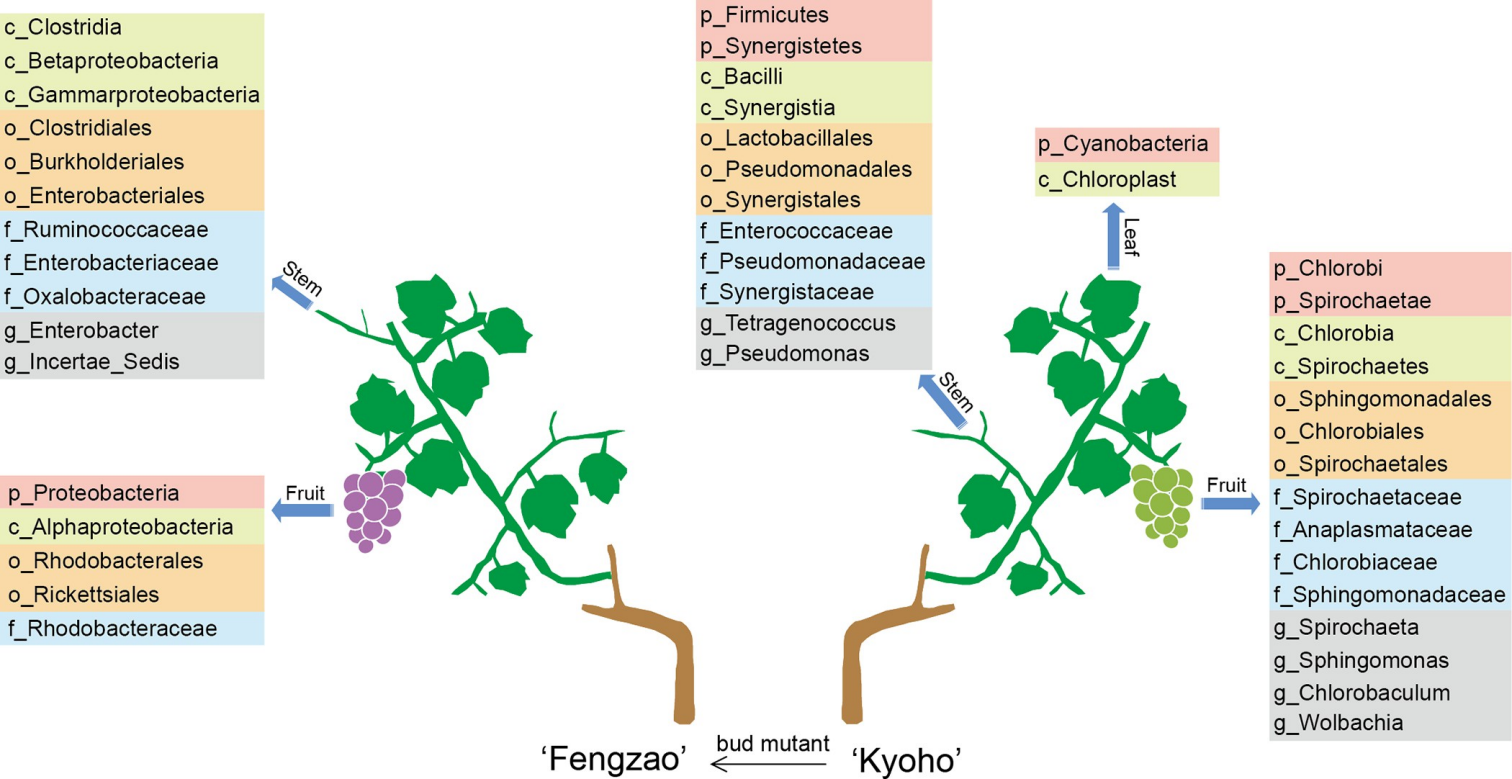
A model map showing the representative microbes in different samples of ‘Fengzao’ and ‘Kyoho’. Only the microbial species with relatively high abundance are shown in the map, based on the results from Fig 2. The different taxonomic levels (p: phylum, c: class, o: order, f: family, g: genus) are indicated with different colored background.

It is worth mentioning that our study revealed the significant variation in microbial composition between grape and its bud mutant variety (Fig 6), which is the first report in horticultural plants to explore microbial community’s variation in bud mutant variety. Current studies mainly focused on the microbial diversity among different plant species and different varieties of same plant species. For example, Portillo et al. [9] found differences in the microbial communities of fruit surface between two grape varieties, Grenache and Carignan. Yang et al. [50] compared the microbial communities in the rhizosphere soils from two varieties of *Camellia sinensis*, indicating that the composition of bacterial and fungal communities of two tea varieties was remarkably different. Similar results were obtained in tobacco and olive, in which the structure and composition of bacterial and fungal communities in different varieties were different [51,52]. So, from these studies, we can deduce that microbiome is genome- or genotype- specific in plants, which should be further investigated in more plant varieties [53,54], because current researches are still limited and insufficient. Additionally, there is no relevant research concerning the microbial variation in bud mutant, which is mostly occurred in horticultural plants. Accordingly, even though obvious variation was observed between ‘Kyoho’ and ‘Fengzao’ in this study, it is still uncertain whether bud mutant will influence microbial community diversity in all the horticultural plants. Therefore, it is of vital importance to deeply investigate the microbiome in bud mutant varieties in more horticultural plants to reveal the mechanisms underlying the bud mutant-related microbial diversity.

In addition, our study also found that different grape tissues harbored significantly different microbial communities. Fruits and stems were rich in microbes, while leaves contained very few microbes, with almost no microbes detected in ‘Fengzao’ leaves (Fig 6). Of course, the microbes inside the roots should be the most abundant. Previous studies have conducted detailed microbiome studies on grape roots, so our study did not replicate the study of the root. Martins et al. [19] examined the microbiome in the roots, barks, leaves and fruits of grapes, and found that the roots were the most abundant in microbes, followed by barks, fruits and leaves. The unique microbial community variation in root or rhizosphere mainly due to the specificity of the host plants themselves [55]. Root is directed related to the external environment and is most affected by it, and plant activities directly affect the microbial diversity of rhizosphere. Therefore, microbial community in rhizosphere exhibited significant species- or genotype-specific [25]. The tissue specificity of microbial compositions reminds us that a whole understanding of the microbiome for a plant requires analysis of different tissues, and although microbes can move within a plant, their final hosts may be relatively stationary.

After identifying the microbes, we also predicted the functional potential of the microbial community based on the COG pathway enrichment (Fig 5) using PICRUSt2 software. We noticed that the COG pathway with most obviously differences between ‘Kyoho’ and ‘Fengzao’ was ‘translation, ribosomal structure and biogenesis’, which is understandable as many of plant microbes are correlated with the processes of translation, ribosomal structure and biogenesis, and the microbes might influence the grape’s life activities by these processes, leading to the variation between these two varieties. The predicted pathways from fruit, leaf and stem were similar, possibly due to the mobility and transference of microorganisms throughout the whole grapevine. It’s worth noting that some pathways related to grape quality physiology, like ‘energy production and conversion’, ‘carbohydrate transport and metabolism’, and ‘inorgenic ion transport and metabolism’, were found in the COG pathway enrichment, indicating that these identified microbes may play a role in the grape fruit quality. Our previous studies demonstrated the physiological differences between ‘Kyoho’ and ‘Fengzao’ [29,31], which might due to the involvement of these microbes, and these inferences of course are needed to be further explored in the future. Certainly, functional prediction with PICRUSt2 can only be used for hypothesis generation, as criticisms for this software often exist. First, this prediction greatly depends on the existing reference genomes, so some specific functions may be less likely to be identified. Second, the strain-specific functions for the same species can’t be distinguished efficiently [37]. Nevertheless, this software is still the most reliable software available compared to the others, with expanded reference database and more compatible and novel approaches [37].

Many microbes identified in this study have been proved as key regulators in plant growth and development [18,44–46,56]. For example, *Proteobacteria* was found significantly enriched in the fruits of ‘Fengzao’. The phylum of *Proteobacteria* can be further categorized as *Alphaproteobacteria*, *Betaproteobacteria*, *Gammarproteobacteria* and *Deltaproteobacteria*, which were also identified in ‘Fengzao’ with a high abundance in fruits and stems (Fig 6). *Rhodobacteraceae*, one of the major subdivisions of *Alphaproteobacteria* [56], was also identified in the fruits of ‘Fengzao’ with a high abundance (Fig 6). Studies have found that *Proteobacteria* can promote growth in polluted farmland [46]. Interestingly, *Proteobacteria* and its subphylum were only identified and enriched in the fruits and stems of ‘Fengzao’, with a significantly lower abundance in ‘Kyoho’ (Figs 2 and 6), suggesting that bacterial abundance of *Proteobacteria* may have an impact on the differences of fruit development between ‘Fengzao’ and ‘Kyoho’. *Firmicutes* were also the main phyla at the early fruit enlargement stage and in the rhizosphere soil in vineyards [45], which were identified from the stems of ‘Kyoho’ in this study. Zhang et al. [45] found that *Firmicutes* were sensitive to abiotic stresses, especially drought. The *Pseudomonas* was found most significantly enriched in the stems of ‘Kyoho’(Fig 6). The content of *Pseudomonas* was high especially on the surface of wine grapes [44]. The *Pseudomonas* can produce extracellular polysaccharide, which is conducive to the formation of microbial membranes and affects the colonization of microbes on the fruit surface [44]. The *Enterobacteriaceae* family or the *Enterobacter* genus was identified with a significantly higher abundance in the stems of ‘Fengzao’ (Fig 6). The *Enterobacteriaceae* is thought to be beneficial for vineyards, as it can produce glucanases, chitinases and proteases to provide host resistance [18]. Additionally, some other identified microbes, like *Ruminococcaceae* family, *Oxalobacteraceae* family and *Rhodobacteraceae* genus from ‘Fengzao’, *Tetragenococcus* genus, *Sphingomonas* genus and *Chlorobaculum* genus from ‘Kyoho’, can be further investigated to understand their roles in grapevines.

## Conclusion

In this study, we analyzed the microbiomes of the ‘Kyoho’ grape and its bud mutant variety (named ‘Fengzao’), identified a total of 34 OTUs from stems, leaves and fruits. There were obvious differences in the microbial communities between ‘Fengzao’ and ‘Kyoho’. The microbes in different grape tissues also showed remarkable differences, and fruits and stems are the tissues with relatively higher abundance of microbes, while the leaves contained less microbes. *Proteobacteria* phylum, *Enterobacteriaceae* family and *Rhodobacteraceae* were abundant in ‘Fengzao’, and *Firmicutes* phylum, *Pseudomonas* genus, family of *Spirochaetaceae*, *Anaplasmataceae*, *Chlorobiaceae*, and *Sphingomonadaceae*, genera of *Spirochaeta*, *Sphingomonas*, *Chlorobaculum* and *Wolbachia* were abundant in ‘Kyoho’. This study first demonstrated the microbial community’s variation between grapevine and its bud mutant variety, and these identified microbes will be significant resources for the future researches on the grapevine microbiology.

## Supporting information

S1 File S1 Table. Statistics of sequencing data. S2 Table. Sequences of each representative OTU. S1 Fig. The length distribution of obtained clean tags in each sample (A: KF; B: FF; C: KL; D: FL; E: KS; F: FS). S2 Fig. Distribution of microorganism species at all levels of phylum (A), class (B), order (C), and family (D). S3 Fig. The Rarefaction curves (A) and the Shannon curves (B) of each sample. S4 Fig. Analysis of COG metabolic pathways in leaves between ‘Fengzao’ (FL) and ‘Kyoho’ (KL). S5 Fig. Analysis of COG metabolic pathways in stems between ‘Fengzao’ (FS) and ‘Kyoho’ (KS).
(ZIP)
