# Supplementary material for: Comparative microbiome analysis reveals the variation in microbial communities between ‘Kyoho’ grape and its bud mutant variety
Source: PLoS One. 2023 Aug 30;18(8):e0290853. doi: 10.1371/journal.pone.0290853 (PMC10468054; doi:10.1371/journal.pone.0290853)
Supplement: S1 File — S1 Table. Statistics of sequencing data. S2 Table. Sequences of each representative OTU. S1 Fig. The length distribution of obtained clean tags in each sample (A: KF; B: FF; C: KL; D: FL; E: KS; F: FS). S2 Fig. Distribution of microorganism species at all levels of phylum (A), class (B), order (C), and family (D). S3 Fig. The Rarefaction curves (A) and the Shannon curves (B) of each sample. S4 Fig. Analysis of COG metabolic pathways in leaves between ‘Fengzao’ (FL) and ‘Kyoho’ (KL). S5 Fig. Analysis of COG metabolic pathways in stems between ‘Fengzao’ (FS) and ‘Kyoho’ (KS). (ZIP) [file pone.0290853.s001.zip › Supporting information/S1 Table.docx]

**S1 Table. Statistics of sequencing data.**

| Sample_ID | PE_Reads | Raw_Tags | Clean_Tags | AvgLen (bp) | GC (%) | Q20 (%) | Q30 (%) | Effective (%) |
| --- | --- | --- | --- | --- | --- | --- | --- | --- |
| FF | 289872 | 260636 | 242231 | 446 | 55.49 | 97.15 | 94.36 | 83.56 |
| FL | 251783 | 226456 | 210727 | 446 | 55.48 | 97.16 | 94.4 | 83.69 |
| FS | 228605 | 204996 | 190359 | 446 | 55.5 | 97.13 | 94.32 | 83.27 |
| KF | 271963 | 240929 | 222759 | 446 | 55.47 | 97.02 | 94.17 | 81.91 |
| KL | 182379 | 165342 | 154073 | 446 | 55.46 | 97.15 | 94.37 | 84.48 |
| KS | 165882 | 145536 | 134281 | 446 | 55.48 | 96.96 | 94.07 | 80.95 |

(Sample_ID: sample name; FF: ‘Fengzao’ fruit; FL: ‘Fengzao’ leaf; FS: ‘Fengzao’ shoot; KF: ‘Kyoho’ fruit; KL: ‘Kyoho’ leaf; KS: ‘Kyoho’ shoot. PE_Reads: number of two-end reads obtained by sequencing; Raw_Tags: number of original sequences obtained by splicing two-end reads; Clean_Tags: The number of optimized sequences obtained after the original sequences are filtered; AvgLen (bp): average sequence length of sample; GC (%): GC content of the sample, that is, the percentage of G and C bases in the total base; Q20 (%): the percentage of bases with mass value greater than or equal to 20 in the total number of bases; Q30 (%): the percentage of bases with mass value greater than or equal to 30 in the total number of bases; Effective(%): percentage of Clean_Tags to PE_Reads.)
