# Supplementary material for: Comparative microbiome analysis reveals the variation in microbial communities between ‘Kyoho’ grape and its bud mutant variety
Source: PLoS One. 2023 Aug 30;18(8):e0290853. doi: 10.1371/journal.pone.0290853 (PMC10468054; doi:10.1371/journal.pone.0290853)
Supplement: S1 File — S1 Table. Statistics of sequencing data. S2 Table. Sequences of each representative OTU. S1 Fig. The length distribution of obtained clean tags in each sample (A: KF; B: FF; C: KL; D: FL; E: KS; F: FS). S2 Fig. Distribution of microorganism species at all levels of phylum (A), class (B), order (C), and family (D). S3 Fig. The Rarefaction curves (A) and the Shannon curves (B) of each sample. S4 Fig. Analysis of COG metabolic pathways in leaves between ‘Fengzao’ (FL) and ‘Kyoho’ (KL). S5 Fig. Analysis of COG metabolic pathways in stems between ‘Fengzao’ (FS) and ‘Kyoho’ (KS). (ZIP) [file pone.0290853.s001.zip › Supporting information/S2 Table.docx]

**S2 Table. Sequences of each representative OTU**

| **No.** | **OTU** | **Sequence** |
| --- | --- | --- |
| 1 | OTU1418 FF_26967 | ACTCCTACGGGAGGCAGCAGTGGGGAATTTTTTGCAATGGGCAAAAGCCCGATGGAGCAATGCCATGTGGAGGTAGAAGGCCCACGGGTCATGAACTTCTTTTCTCGAAGAAGAAACAATGACAATATCTGGGGAATAAGCATCGACTAACTTTATGCCAGCAACCATGGTAATACAGAGGATGCAAGCGTTATCTGGAATGATTGGGTGTAAAACGTCAGTAGGTGGCTTTTTAAGTCCGCCTTCAAATCCCAAGGCTCAACCCTAGACAAGCAATGGAAACTACCAAGCTAGAATACGGTAGGGGTAGAGGGAATTTATGATAGAGTAGTGAAATGCGTAGAGATTGGAAAGAATACCAACGACAAGAGCACTCTACGGGCTGACACTAACACTGAGAGAAGAAAATTAGGGGAGCGAATGGGATTAGAAACCCTAGTAGTCC |
| 2 | OTU1664 FF_20314 | ACTCCTACGGGAGGCAGCAGTGGGGAATTTTCCGCAATGGGCGAAAGCCTGACGGAGCAATGCCGCGTGGAGGTAGAAGGCCCACGGGTCGTGAACTTCTTTTCCCGGAGAAGAAGCAATGACGGTATCTGGGGAATAAGCATCGGCTAACTCTGTGCCAGCAGCCGCGGTAAGACGGGGGGGGCAAGTGTTCTTCGGAATGACTGGGCGTAAAGGGCACGTAGGCGGTGAATCGGGTTGAAAGTGAAAGTCGCCAAAAAGTGGCGGAATGCTCTCGAAACCAATTCACTTGAGTGAGACAGAGGAGAGTGGAATTTCGTGTGTAGGGGTGAAATCCGGAGATCTACGAAGGAACGCCAAAAGCGAAGGCAGCTCTCTGGGTCCCTACCGACGCTGGGGTGCGAAAGCATGGGGAGCAAACGGGATTAGAAACCCTAGTAGTCC |
| 3 | OTU2296 FL_77149 | ACTCCTACGGGAGGCAGCAGCTAAGAATATTCCGCAATGGACGGAAGTCTGACGGAGCGACGCCGCGTGGATGACGAAGGCCGAAAGGTTGTAAAGTTCTTTTGCTGGGGAAGAATAACCGCAGGAGGGAATGCCTGTGGGATGACAAGAACCGGCGAATAAGCCCCGGCCAACTACGTGCCAGCAGCCGCGGTAACACGTAGGGGGCGAGCGTTGTTCGGAATTACTGGGCGTAAAGGGCATGTAGGCGGCTTGGAAAGCCTGGCGTGAAAGTCTGCAGCTTAACTGCAGGGGTGCGCTGGGAACTGCTGAGCTTGAGTGACGGAGAGGGAGCTGGAATTCCTGGTGTAGGGGTGAAATCTGTAGATATCAGGAAGAACACCGATGGCGAAGGCAAGCTCCTGGCCGATGACTGACGCTGAGGTGCGAAAGTGTGGGGATCAAACAGGATTAGAAACCCCAGTAGTCC |
| 4 | OTU2554 FF_153152 | ACTCCTACGGGAGGCAGCAGTGGGGAATCTTGGACAATGGGCGAAAGCCCGATCCAGCAATATCGCGTGAGTGAAGAAGGGCAATGCCGCTTGTAAAGCTCTTTCGTCGAGTGCGCGATCATGACAGGACTCGAGGAAGAAGCCCCGGCTAACTCCGTGCCAGCAGCCGCGGTAATACAGAGGATGCAAGCGTTATCCGGAATGATTGGGCGTAAAGCGTCTGTAGGTGGCTTTTTAAGTCCGCCGTCAAATCCCAGGGCTCAACCCTGGACAGGCGGTGGAAACTACCAAGCTGGAGTACGGTAGGGGCAGAGGGAATTTCCGGTGGAGCGGTGAAATGCGTAGAGATCGGAAAGAACACCAACGGCGAAAGCACTCTGCTGGGCCGACACTGACACTGAGAGACGAAAGCTAGGGGAGCGAATGGGATTAGAAACCCTAGTAGTCC |
| 5 | OTU2622 FF_12696 | ACTCCTACGGGAGGCAGCAGTGGGGAATATTGGACAATGGGCGCAAGCCTGATCCAGCCATGCCGCGTGAGTGATGAAGGCCCTAGGGTTGTAAAGCTCTTTCACCGGTGAAGATAATGACGGTAACCGGAGAAGAAGCCCCGGCTAACTTCGTGCCAGCAGCCGCGGTAATACGAAGGGGGCTAGCGTTGTTCGGATTTACTGGGCGTAAAGCGCACGTAGGCGGACTTTTAAGTCAGGGGTGAAATCCCGGGGCTCAACCCCGGAACTGCCTTTGATACTGGAAGTCTTGAGTATGGTAGAGGTGAGTGGAATTCCGAGTGTAGAGGTGAAATTCGTAGATATTCGGAGGAACACCAGTGGCGAAGGCGGCTCACTGGACCATTACTGACGCTGAGGTGCGAAAGCGTGGGGAGCAAACAGGATTAGAAACCCTAGTAGTCC |
| 6 | OTU2892 FS_52480 | ACTCCTACGGGAGGCAGCAGTGGGGAATTTTCCGCAATGGGCGAAAGCCTGACGGAGCAATGCCGCGTGGAGGTAGAAGGCCCACGGGTCGTGAACTTCTTTTCCCGGAGAAGAAGCAATGACGGTATCTGGGGAATAAGCATCGGCTAACTCTGTGCCAGCAGCCGCGGTAATACAGAGGATGCAAGCGTTATCCGGAATGATTGGGCGTAAAGCGTCTGTAGGTGGCTTTTTAAGTCCGCCGTCAAATCCCAGGGCTCAACCCTGGACAGGCGGTGGAAACTACCAAGCTGGAGTACGGTAGGGGCAGAGGGAATTTCCGGTGGAGCGGTGAAATGCGTAGAGATCGGAAAGAACACCAACGGCGAAGGCAGCTCTCTGGGTCCCTACCGACGCTGGGGTGCGAAAGCATGGGGAGCAAACGGGATTAGAAACCCTTGTAGTCC |
| 7 | OTU2939 FL_165832 | ACTCCTACGGGAGGCAGCAGTGGGGAATTTTCCGCAATGGGCGAAAGCCTGACGGAGCAATGCCGCGTGGAGGTAGAAGGCCCACGGGTCGTGAACTTCTTTTCCCGGAGAAGAAGCAATGATGGTATCTGGGGAATAAGCATCGGCTAACTCTGTGCCAGCCGCCGCGGTAATACAGAGGATGCAAGCGTTATCCGGAATGATTCGGCGTAAAGCGTCAGTAGCTGGCTTTTTAAGTCCGCCGTCAAATCCCAGTGCTGAACCCTGGACAGGCGGTGGAAACTACCAATCTGGAGTACGGTAGGGGCAGAGGGAATTTCCCGTGGAGCGGTGAAATGCGTAGAGATCGGACAGTACACCAACCGCGAAAGCACTCTGCTGGGCCGCCACTGACACTTAGAGACGAAAGCTAGGGGAGCGAATGGGATTAGATACCCTAGTAGTCC |
| 8 | OTU3079 FF_1149 | ACTCCTACGGGAGGCAGCAGTGGGGAATATTGGACAATGGGCGCAAGCCTGATCCAGCCATGCCGCGTGAGTGATGAAGGCCCTAGGGTTGTAAAGCTCTTTCAGCGAGGAGGATAATGACGTTACTCGCAGAAGAAGCCCCGGCTAACTTCGTGCCAGCAGCCGCGGTAATACGAAGGGGGCTAGCGTTGTTCGGAATCACTGGGCGTAAAGCGCACGTAGGCGGACTTTTAAGTCAGGGGTGAAATCCCGGGGCTCAACCTCGGAACTGCCTTTGATACTGGAAGTCTGGAGTCCGAGAGAGGTGAGTGGAACTCCGAGTGTAGAGGTGAAATTCGTAGATATTCGGAAGAACACCAGTGGCGAAGGCGGCTCACTGGCTCGGTACTGACGCTGAGGTGCGAAAGCGTGGGGAGCAAACAGGATTAGAAACCCTAGTAGTCC |
| 9 | OTU3218 KL_83032 | ACTGGTACGGGAGGCAGCAGTGGGGAATTTTCCGCAATGGGCGAAAGCCTGACGGAGCAATGCCGCGTGGAGGTAGAAGGCCCACGGGTCGTGAACTTCTTTTCCCGGAGAAGAAGCAATGACGGTATCTGGGGAATAAGCATCGGCTAACTCTGTGCCAGCAGCCGCGGTAATACAGAGGATGCAAGCGTTATCCGGAATGATTGGGCGTAAAGCGTCTGTAGGTGGCTTTTTAAGTCCGCCGTCAAATCCACGGGCTCAACCCTGGTCAGGCGGGCGAAACTATCAAGCTGGAGTACGGTAGGGGCAGATGGAATTTCCGGAGGAGCGGTGAAATGCGTCGAGATCGGAAAGAACACCTACGGCGGAAGCACTCTGCTGGGCCGACACTGACACTGAGAGACGAAAGCTAGGGGAGCGAATGGGATTAGAAACCCTGGTAGTCC |
| 10 | OTU3488 KF_3792 | ACTCCTACGGGAGGCAGCAGTGGGGAATATTGGACAATGGGCGAAAGCCTGATCCAGCCATGCCGCATGAGTGAAGAAGGCCTTTGGGTTGTAAAGCTCTTTTAGTGAGGGAGATAATGACGGTACTCACAGAAGAAGTCCTGGCTAACTCCGTGCCAGCAGCCGCGGTAATACGGAGAGGGCTAGCGTTATTCGGAATTATTGGGCGTAAAGGGCGCGTAGGCTGGTTAATAAGTTAAAAGTGAAATCCCGAGGCTTAACCTTGGAATTGCTTTTAAAACTATTAATCTAGAGATTGAAAGAGGATAGAGGAATTCCTGATGTAGAGGTAAAATTCGTAAATATTAGGAGGAACACCAGTGGCGAAGGCGTCTATCTGGTTCAAATCTGACGCTGAGGCGCGAAGGCGTGGGGAGCAAACAGGATTAGAAACCCTAGTAGTCC |
| 11 | OTU3613 KF_178715 | ATTCCTACGGGAGGCAGCAGTGGGGAATTTTCCGCAATGGGCGAAAGCCTGACGGAGCAATGCCGCGTGGAGGTAGAAGGCCCACGGGTCGTGAACTTCTTTTCCCGGAGAAGAAGCAATGACGGTATCTGGGGAATAAGCATCGGCTAACTCTGTGCCAGCGGCCGCGGTAATGCACAGGACGCAAGCGTTATCCGGAATGATTGGGCGTAAAGCGTCTGTAGGTGGCTTTTTAAGTCCGCCGTCAAATCCTAGGTCTCAACCCTGGACAGGCGGTGGAAACTACCAAGCTGGAGTACGGTAGGGGCAGAGGGAATTTCCGGTGGAGCGGTGAAATTCGTATAGCTCGGACAGAACACCAACGGCGAAAGCACTCTGCTGGGCCGACACTGACACTGAGAGACGAAAGCTAGGGGAGCGAATGGGATTAGATATCCCTGTAGTCC |
| 12 | OTU3748 FS_64371 | ACTCCTACGGGAGGCAGCAGTGAGGAATATTGCGCAATGGGCGAAAGCCTGACGCAGCAACGCCGCGTGGATGATGAAGTTCTTCGGAATGTAAAATCCTTTTGCAGGGGACGAATAGGTCGGCTTGCCGACTCTGACGGTACCCTGCGAATAAGCCACGGCTAACTCTGTGCCAGCAGCCGCGGTGATACAGGGGTGGCAAGCGTTGTCCGGATTTACTGGGTGTAAAGGGTGCGCAGGCGGATCAATAAGTCGGGGGTTAAATCCATGTGCTTAACACATGCACGGCTTCCGATACTGTTGATCTAGAGTCTCGAAGAGGAAGGTGGAATTTCCGGTGTAACGGTGGAATGTGTAGATATCGGAAAGAACACCAGTGGCGAAGGCAGCCTTCTGGTCGAGTACTGACGCTCATGCACGAAAGCGTGGGGAGCAAACAGGATTAGAAACCCTAGTAGTCC |
| 13 | OTU4063 FS_110266 | ACTCCTACGGGAGGCAGCAGTGGGGAATTTTCCGCAATGGGCGAAAGCCTGACGGAGCAATGCCGCGTGGAGGTAGAAGGCCCACGGGTCGTGAATTTCTTTTCCCGGAGAAGAAGCAATGACGGTATCTGGGGAATAAGGATCGGCTGACTCTGAGCCAGCAGCCGCGGTAATACAGAGGATGCATCCGATATACGGAATGATTGGGCGTAAAGCGTCGGTAGGTGGCTTTTTAAGTCCGCCGTCAAATCCCAGGGCTTAACCCTGGACAGGCGGTGGAAACTACCAAGCTGTAGTACGGTAGGGGCAGAGGGAATTTCCGGTGGAGCGGTGAAATGCGTAGAGATCGGAAAGAACACCAACGGCGAAAGCACTCTGCTGGGACGACACTGACACTGAGAGACGAAAGCTAGGGGAGCGAATGGTATTAGAAACCCTGGTAGTCC |
| 14 | OTU4313 FF_12800 | ACTCCTACGGGAGGCAGCAGTAGGGAATCTTCGGCAATGGACGCAAGTCTGACCGAGCAACGCCGCGTGAGTGAAGAAGGTTTTCGGATCGTAAAGCTCTGTTGTCAGCAAAGAACAGGAGAAAGAGGCAATGCTTTTTCTATGACGGTAGCTGACCAGAAAGCCACGGCTAACTACGTGCCAGCAGCCGCGGTAATACGTAGGTGGCAAGCGTTGTCCGGATTTATTGGGCGTAAAGCGAGCGCAGGCGGTGATTTAAGTCTGATGTGAAAGCCCCCAGCTCAACTGGGGAGGGTCATTGGAAACTGGATCACTTGAGTGCAGAAGAGGAGAGTGGAATTCCATGTGTAGCGGTGAAATGCGTAGATATATGGAGGAACACCAGTGGCGAAGGCGGCTCTCTGGTCTGTAACTGACGCTGAGGCTCGAAAGCGTGGGTAGCAAACAGGATTAGAAACCCTAGTAGTCC |
| 15 | OTU4701 FF_19626 | ACTCCTACGGGAGGCAGCAGTGGGGAATATTGGACAATGGGCGAAAGCCTGATCCAGCAATGCCGCGTGAGTGATGAAGGCCTTAGGGTTGTAAAGCTCTTTTACCCGGGATGATAATGACAGTACCGGGAGAATAAGCTCCGGCTAACTCCGTGCCAGCAGCCGCGGTAATACGGAGGGAGCTAGCGTTATTCGGAATTACTGGGCGTAAAGCGCACGTAGGCGGCTTTGTAAGTTAGAGGTGAAAGCCTGGAGCTCAACTCCAGAATTGCCTTTAAGACTGCATCGCTTGAATCCAGGAGAGGTGAGTGGAATTCCGAGTGTAGAGGTGAAATTCGTAGATATTCGGAAGAACACCAGTGGCGAAGGCGGCTCACTGGACTGGTATTGACGCTGAGGTGCGAAAGCGTGGGGAGCAAACAGGATTAGAAACCCTAGTAGTCC |
| 16 | OTU4736 FF_124327 | ATTCCTACGGGAGGCAGCAGTGGGGAATTTTCCGCAATGGGCGAAAGCCTGACGGAGCACTGCCGCGTGGAGGTAGAAGGCCCACGGGTCGTGAACTTCTTTTCCCGGAGAAGAAGCAATGACGGTATCTGGGGAATAAGCATCGGCTAACTCTGTGCCAGCAGCCGCGGTAATACAGAGGATGCAAGCGTTATCCGGAATGATTGGGCGTAAAGGGCACGTAGGCGGTGAATCGGGTTGAAAGTGAAAGTCGCCAAAAAGTGGCGGAATGCTCTCGAAAGCAATTCACTTGAGTGAGACAGAGGAGAGTGGTATTTCGTGTGTAGGGGTGAAATCCGGAGATCTACGAAGGAACGCCAAAAGCGAAGGCAGCTCTCTGGGTCCCTACCGACGCTGGGGTGCGAAAGCATGGGGAGCAAACGGGATTAGATACCCGAGTAGTCC |
| 17 | OTU477 FL_81033 | ACTCCTACGGGAGGCAGCAGCTAAGAATATTCCGCAATGGACGAAAGTCTGACGGAGCGACGCCGCGTGGATGATGAAGGCCGAAAGGTTGTAAAGTCCTTTTGTCGACGAAGAATAAGTGTGGGAGGGAATGCCCGCATGATGACGTTAGTCGGCGAATAAGCCCCGGCTAATTACGTGCCAGCAGCCGCGGTAACACGTAAGGGGCGAGCGTTGTTCGGAATTATTGGGCGTAAAGGGTACGTAGGCGGTTTGGTAAGCCCGGCGTGAAATACTGGAGCTCAACTCCAGAACTGCGTTGGGAACTGCGAGACTTGAGTCATGGAAGGGGAGTTGGAATTCCAGGTGTAGGGGTGAAATCTGTAGATATCTGGAAGAACACCGGTGGCGAAGGCGAACTCCTGGCCAATGACTGACGCTGAGGTACGAAAGTGCGGGGAGCAAACAGGATTAGAAACCCTAGTAGTCC |
| 18 | OTU5662 FL_121804 | ACTCCTACGGGAGGCAGCAGTGGGGAATTTTGCGCAATGGGCGAAAGCCTGACGGAGCAATGCCGCGTGGAGGTAGAAGGCCCACGGGTCGTTAACTTCTTTTCCCGGAGAAGAAGCAATGACGGTATCTGGGGAATAAGCATCTGCTGACTCTGAGCCAGCAGCCGCGGTAATACAGAGGATGCAGTCGATATGCGGAATGATTGGGCGTAAAGCGTCTGTAGGTGGCTTTTTAAGTCCGCCGTCAAATCCCAGGGATCAACCCTGGACAGGCGGTGGAAACTACCAAGCTGCAGTACGGTAGGGGCAGAGGGAATTTCCGGTGGAGCGGTGAAATGCGTAGAGATCGGAAAGAACACCAACGGCGAAAGCACTCTGCTGGGTCGACACTGACACTGAGAGACGAAAGCTAGGGGAGCGAATGGGATTAGATACCCGAGTAGTCC |
| 19 | OTU5889 FS_80381 | ACTCCTACGGGAGGCAGCAGTGGGGAATATTGGGCAATGGGCGCAAGCCTGACCCAGCGACGCCGCGTGGGGGAAGAAGTCTTTCGGGACGTAAACCCATGTTGTACGGGACGAAGGAAGTGACGGTACCGTACGAGGAAGCCCCGGCAAACTACGTGCCAGCAGCCGCGGTAATACGTAGGGGGCGAGCGTTGTCCGGAATCACTGGGCGTAAAGCGCACGTAGGCGGGCTGTTAAGTCGGCCGTGAAATACACTGGCTCAACCGGTGCAGGTCGGTCGATACTGGCGGTCTGGAGTATGGGAGAGGGAACTGGAATTCCCGGTGTAGCGGTGAAATGCGTAGATATCGGGAGGAACACCAGTGGCGAAGGCGGGTTCCTGGCCCATGACTGACGCTGAGGTGCGAAAGCCGGGGGAGCGAACGGGATTAGATACCCCAGTAGTCC |
| 20 | OTU6041 FF_133482 | ACTCCTACGGGAGGCAGCAGTGGGGAATTTTCCGCAATGGGCGAAAGCCTGACGGAGCAATGCCGCGTGGAGGTAGAAGGCCCACGGGTCGTGAACTTCTTTTCCCGGAGAAGAAGCAATGACGGTATCTGGGGAATAAGCATCGGCTAACTCTGTGCCAGCAGCCGCGGTAATACAGAGGATGCAAGCGTTAGCCGGAGTGATTGGGCGTAAAGCGTCTGTAGGTGGCTTTTTAAGTCCGCCGTCAAACACCAGGGCTCAACCCTGTACAGGCGGTGGAAACTGCCAAGCTGGAGTACGGTAGGGGCAGAGGGCATCTCCGATGGATCGGTGAAATGCGTAGAGATCGGAAAGAACACCAACGGCGAAAGCACTCTGCTGGGCCGACACTGACACTGCGAGACGAAAGCTAGGGGAGCGATTGGGATTAGAAACCCCGGTAGTCC |
| 21 | OTU623 FL_145217 | ACTCCTACTTTAGTCATCATTTGTGAATTTTCCGCAATGTGCGAAAGCCTTACGGAGCAATGCCTCGTGGAGTTAGAAGGCCCACGGGTCGTGAACTTCTTTTCCCGGATAAGAAGCAATGACGGTATCTGGGGAATAAGCATCGGCTAACTCTGTGCCAGCAGCCGCGGTAATACAGAGGATGCAAGCGTTATCCGGAATGATTGGGCGTAAAGCGTCTGTAGGTGGCTTTTTAAGTCCGCCGTCAAATCACAGGGCTCACACCTGGACAGGCGGTGGAAACTACCAAGCTGGAGTACGGTAGGGGCAGAGGGAATTTACGGTGGAGCGGTGAAATGCGTAGAGATCGGAAAGAACACCAACGGCGAAAGCACTCTGCTGGGCCGACACTGACACTGAGAGACGAAAGCTAGGGGAGCGAATGGGATTAGAAACCCTAGTAGTCC |
| 22 | OTU6515 KF_5095 | ACTTCTACGGGAGGCAGCAGTGGGGAATTTTCCGCAATGGGCGAAAGCCTGACGGAGCAATGCCGCTTGTAAAGCTCTTTCGTCGAGTGCGCGATCATGACAGGACTCGAGGAAGAAGCCCCGGCTAACTCCGTGCCAGCAGCCGCGGTAAGACGGGGGGGGCAAGTGTTCTTCGGAATGACTGGGCGTAAAGGGCACGTAGGCGGTGAAGCGGGTTGAAAGTGAAAGTCGCCAAAAAGTGGCGGAATGCTCTCGAAACCAATTGACTTGAGTGAGACAGAGGAGAGTGGAATTTCGTGTGTAGGGGTGAAATCCGGAGATCTACGAAGGAACGCCAAAAGCGAAGGCAGCTCTCTGGGTCCCTACCGACGCTGGGGTGCGAAAGCATGGGGAGCAAACGGGATTAGAAACCCCGGTAGTCC |
| 23 | OTU6717 FS_123179 | ACTCCTACTTTAGTCATCATTTTTGAATTTTCCGCAATTTTCTAAAGCCTTACGGAGCAATTCCTCTTTGAGTTAGAATTCCCACGTGTCGTGAACTTCTTTTCCCGGATAAGAAGCAATGACGGTATCTGGGGAATAAGCATCGGCTAACTCTGTGCCAGCAGCCGCGGTAATACAGAGGATGCAAGCGTTATCCGGAATGATTGGGCGTAAAGCGTCTGTAGGTGGCTTTTTAAGTCCGCCGTCAAATCACAGGGCTCAAACCTGGACAGGCGGTGGAAACTACCAAGCTGGAGTACGGTAGGGGCAGAGGGAATTTACGGTGGAGCGGTGAAATGAGTAGAGATCGGAAAGAACACCAACGGCGAAAGCACTCTGCTGGGCCGACACTGACACTGAGAGACGAAAGATAGGGGAGCGAATGGGATTAGATACCCTAGTAGTCC |
| 24 | OTU6854 FL_46257 | ACTCCTACGGGAGGCAGCAGTGGGGAATATTGCGCAATGGGGGAAACCCTGACGCAGCAACGCCGCGTGAAGGATGAAGGTTTTCGGATCGTAAACTTCTTTGATGAGGGAAGAAAAATGACGGTACCTCAAAAACAAGCCACGGCTAACTACGTGCCAGCAGCCGCGGTAATACGTAGGTGGCGAGCGTTGTCCGGATTTACTGGGTGTAAAGGGCGTGTAGGCGGGGAAGCAAGTCAGATGTGAAACTCCGAGGCTTAACTTCGGAACTGCATCTGAAACTGCATCTCTTGAGTGCTGGAGAGGATAGCGGAATTCCTAGTGTAGCGGTGAAATGCGTAGATATTAGGAGGAACACCAGTGGCGAAGGCGGCTATCTGGACAGTAACTGACGCTGAGGCGCGAAAGCGTGGGGAGCAAACAGGATTAGAAACCCTGGTAGTCC |
| 25 | OTU6916 FF_155860 | ACTTCTACTTGAGTCATCATTGTGGAATTTTCCGCAATGTGCGAAAGCCTGACGGAGCAATTCCGCGTGGAGTTAGAAGGCCCACGTGTCGTGAACTTCTTTTCCCGGAGAAGAAGCAATGACGGTATCTGGGGAATAAGCATCGGCTAACTCTGTGCCAGCAGCCGCGGTAATACAGAGGATGCAAGCGTTATCCGGAATGATTGGGCGTAAAGCGTCTGTAGGTGGCTTTTTAAGTCCGCCGTCAAATCACAGGGCTCAAACCTGGACAGGCGGTGGAAACTACCAAGCTGGAGTACGGTAGGGGCAGAGGGAATTTCCGGTGGAGCGGTGAAATGCGTAGAGATCGGAAAGAACACCAACGGCGAAAGCACTCTGCTGGGCCGACACTGACACTGAGGGACGAAAGATAGGGGAGCGAATGGGATTAGAAACCCTAGTAGTCC |
| 26 | OTU6922 KF_107243 | ACTCCTACGGGAGGCAGCAGTGGGTAATTTTGCGCAATGGTCGAAAGCCTGACGGAGCAATGCCGCTTGGAGTTAGAAGTCCCACGTGTCGTGAACTTCTTTTCCCGGATAAGAAGCAATGACGGTATCTGGGGAATAAGCATCGGCTAACTCTGTGCCAGCAGCCGCGGTAATACAGAGGATGCAAGCGTTATCCGGAATGATTGGGCGTAAAGCGTCGTTAGGTGGCTTTTTAAGTCCGCCGTCAAATCCCAGGGCTCAACCCTGGACAGGCGGTGGAAACTACCAAGCTGGAGTACGGTAGGGGCAGAGGGAATTTCCGGTGGAGCGGTGCCATGCGTAGAGATCGGAAAGAACACCAACGGCGCAAGCACTCTGCTGGGCCGACACTGACACTGAGAGACGAAAGCTAGGGGAGCGAATGGGATTAGATACCCGAGTAGTCC |
| 27 | OTU7563 FS_138094 | ACTCCTACGGGAGGCAGCAGTGGGGAATATTGGACAATGGGCGAAAGCCTGATCCAGCCATGCCGCGTGTGTGAAGAAGGCCTTCGGGTCGTAAAGCACTTTAAGTTGGGAGGAAGGGCTCATAGCGAATACCTGTGAGTTTTGACGTTACCAACAGAATAAGCACCGGCTAACTTCGTGCCAGCAGCCGCGGTAATACGAAGGGTGCAAGCGTTAATCGGAATTACTGGGCGTAAAGCGCGCGTAGGTGGCTTGATAAGTTGGATGTGAAATCCCCGGGCTCAACCTGGGAACTGCATCCAAAACTGTCTGGCTAGAGTGCGGTAGAGGGTAGTGGAATTTCCAGTGTAGCGGTGAAATGCGTAGATATTGGAAGGAACACCAGTGGCGAAGGCGACTACCTGGACTGACACTGACACTGAGGTGCGAAAGCGTGGGGAGCAAACAGGATTAGAAACCCTAGTAGTCC |
| 28 | OTU7879 FS_643 | ACTCCTACGGGAGGCAGCAGTGGGGAATATTGCACAATGGGCGCAAGCCTGATGCAGCCATGCCGCGTGTATGAAGAAGGCCTTCGGGTTGTAAAGTACTTTCAGCGGGGAGGAAGGTGTTGTGGTTAATAACCGCAGCAATTGACGTTACCCGCAGAAGAAGCACCGGCTAACTCCGTGCCAGCAGCCGCGGTAATACGGAGGGTGCAAGCGTTAATCGGAATTACTGGGCGTAAAGCGCACGCAGGCGGTCTGTCAAGTCGGATGTGAAATCCCCGGGCTCAACCTGGGAACTGCATTCGAAACTGGCAGGCTAGAGTCTTGTAGAGGGGGGTAGAATTCCAGGTGTAGCGGTGAAATGCGTAGAGATCTGGAGGAATACCGGTGGCGAAGGCGGCCCCCTGGACAAAGACTGACGCTCAGGTGCGAAAGCGTGGGGAGCAAACAGGATTAGAAACCCTAGTAGTCC |
| 29 | OTU7914 FF_119 | ACTCCTACGGGAGGCAGCAGTGGGGAATCTTGGACAATGGGCGAAAGCCCGATCCAGCAATATCGCGTGAGTGAAGAAGGGCAATGCCGCTTGTAAAGCTCTTTCGTCGAGTGCGCGATCATGACAGGACTCGAGGAAGAAGCCCCGGCTAACTCCGTGCCAGCAGCCGCGGTAAGACGGGGGGGGCAAGTGTTCTTCGGAATGACTGGGCGTAAAGGGCACGTAGGCGGTGAATCGGGTTGAAAGTGAAAGTCGCCAAAAAGTGGCGGAATGCTCTCGAAACCAATTCACTTGAGTGAGACAGAGGAGAGTGGAATTTCGTGTGTAGGGGTGAAATCCGGAGATCTACGAAGGAACGCCAAAAGCGAAGGCAGCTCTCTGGGTCCCTACCGACGCTGGGGTGCGAAAGCATGGGGAGCAAACGGGATTAGAAACCCTAGTAGTCC |
| 30 | OTU8110 FS_65711 | ACTCCTACGGGAGGCAGCAGTGGGGAATTTTGGACAATGGGCGCAAGCCTGATCCAGCAATGCCGCGTGAGTGAAGAAGGCCTTCGGGTTGTAAAGCTCTTTTGTCAGGGAAGAAACGGCTGAGGTTAATACCTTCGGCTAATGACGGTACCTGAAGAATAAGCACCGGCTAACTACGTGCCAGCAGCCGCGGTAATACGTAGGGTGCAAGCGTTAATCGGAATTACTGGGCGTAAAGCGTGCGCAGGCGGTTTTGTAAGTCTGTCGTGAAAGCCCCGGGCTCAACCTGGGAATTGCGATGGAGACTGCAAGGCTTGAATCTGGCAGAGGGGGGTAGAATTCCACGTGTAGCAGTGAAATGCGTAGAGATGTGGAGGAACACCGATGGCGAAGGCAGCCCCCTGGGTCAAGATTGACGCTCATGCACGAAAGCGTGGGGAGCAAACAGGATTAGAAACCCTAGTAGTCC |
| 31 | OTU8121 FF_1 | ACTCCTACGGGAGGCAGCAGTGGGGAATTTTCCGCAATGGGCGAAAGCCTGACGGAGCAATGCCGCGTGGAGGTAGAAGGCCCACGGGTCGTGAACTTCTTTTCCCGGAGAAGAAGCAATGACGGTATCTGGGGAATAAGCATCGGCTAACTCTGTGCCAGCAGCCGCGGTAATACAGAGGATGCAAGCGTTATCCGGAATGATTGGGCGTAAAGCGTCTGTAGGTGGCTTTTTAAGTCCGCCGTCAAATCCCAGGGCTCAACCCTGGACAGGCGGTGGAAACTACCAAGCTGGAGTACGGTAGGGGCAGAGGGAATTTCCGGTGGAGCGGTGAAATGCGTAGAGATCGGAAAGAACACCAACGGCGAAAGCACTCTGCTGGGCCGACACTGACACTGAGAGACGAAAGCTAGGGGAGCGAATGGGATTAGAAACCCTAGTAGTCC |
| 32 | OTU8408 KS_72826 | ACTGGTACGGGAGGCAGCAGTGGGGAATTTTCCGCAATGGGCGAAAGCCTGACGGAGCAATGCCGCGTGGAGGTAGAAGGCCCACGGGTCGTGAACTTCTTTTCCCGGAGAAGAAGCAATGACGGTATCTGGGGAATAAGCATCGGCTAACTCTGTGCCAGCAGCCGCGGTAATACAGAGGATGCAAGCGTTATCCGGAATGATTGGGCGTAAAGCGTCTGTAGGTGGCTTTTTAAGTCCGCCGTCAAATCCCCGGGCTCTATCCTGGGTAGGTGGTGGAAACTACTAAGCTGGAGTTCGGTAGGCGCAGAGGGACTTTCCGGGGGAGCGGTGAAATGCGTCGAGATCGGAAAGAACACCAACGGCGAAAGCACTCTGCTGGGCCGACACTGACACTGAGAGACGAAAGCTAGGGGAGCGAATGGGATTAGAAACCCTAGTAGTCC |
| 33 | OTU8592 KS_111221 | ACTCCTACGGGAGGCAGCAGTGGGGAATTTTCCGCAATGGGCGAAGGCCTGACGGAGCAATGCCGCGTGGAGGTAGAAGGCCCACGGGTCGTGAACTTCTTTTCCGTGAGAAGAAGCAATGAGGGTATCAGGGGAATAAGCATAGGCTAACTCTGTGACAGCAGCCGCGGTAATACAGAGGATGCAAGCGTTATCCGGAATGATTGGGCGTAAAGCGTCTGTAGGTGGCTTTTTAAGTCTGCCGTCAAATCCCAGGGCTCAACCCGTGACAGGCGGTGGAAACTACCAAGCTGGAGTACGGTAGGGGCAGAGGGAATTTCCGGTGGAGCGGTGAAATGCGTAGAGATCGGAAAGAACACCAACGGCGAAAGCACTCTGCTGGGCCGACACTGACACTGAGAGACGAAAGCTAGGCGCGCGAATGGGATTAGAAACACCAGTAGTCC |
| 34 | OTU893 FS_107798 | ACTTCTACGGGAGGCAGCAGTGGGGAATTTTCCGCAATGGGCGAAAGCCTGACGGAGCAATGCCGCGTGGAGGTAGAAGGCACACGGGTCGTGAACTTCTTTTCCCGGAGAAGAAGCAATGACGGTATCTGGGGAATAAGCATCGGCTAACTCTGTGCCAGCAGCCGTGGTAATACAGAGGATGGAAGCGTTCTCCGGAATGATTGGGTGTAAAGCGTCTGTAGGTGGCTTTTTAAGTCCGCCGTCAAATCCCAGGGCTCAACCCTGGACCGGCGGTGGACACTACCAAGCTGGAGTACGGTAGGGGCAGAGGGAATTTACGGTGGAGTCGGCAAATGCGTAGTGATCGGAAAGAACACCAACGGCGAAAGCACTCTGCTGGGCCGACACTGACACTGAGAGACGAAAGCTAGGGGAGCGAATGGGATTAGAAACCCCAGTAGTCC |
