# Supplementary figures and images for: Comparative microbiome analysis reveals the variation in microbial communities between ‘Kyoho’ grape and its bud mutant variety
Source: PLoS One. 2023 Aug 30;18(8):e0290853. doi: 10.1371/journal.pone.0290853 (PMC10468054; doi:10.1371/journal.pone.0290853)

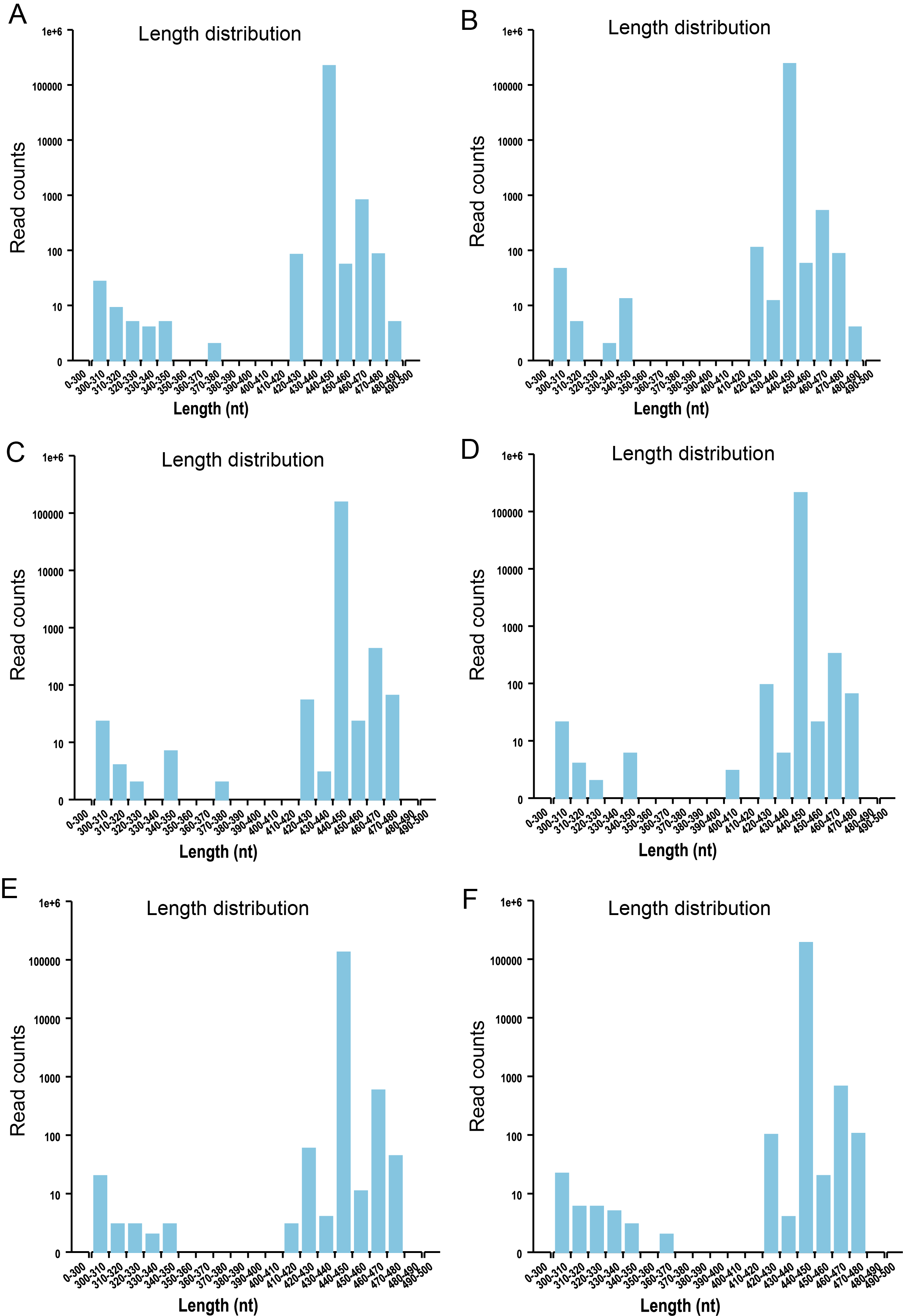

Supplement: S1 File — S1 Table. Statistics of sequencing data. S2 Table. Sequences of each representative OTU. S1 Fig. The length distribution of obtained clean tags in each sample (A: KF; B: FF; C: KL; D: FL; E: KS; F: FS). S2 Fig. Distribution of microorganism species at all levels of phylum (A), class (B), order (C), and family (D). S3 Fig. The Rarefaction curves (A) and the Shannon curves (B) of each sample. S4 Fig. Analysis of COG metabolic pathways in leaves between ‘Fengzao’ (FL) and ‘Kyoho’ (KL). S5 Fig. Analysis of COG metabolic pathways in stems between ‘Fengzao’ (FS) and ‘Kyoho’ (KS). (ZIP) [file pone.0290853.s001.zip › Supporting information/S1 Fig.tif]

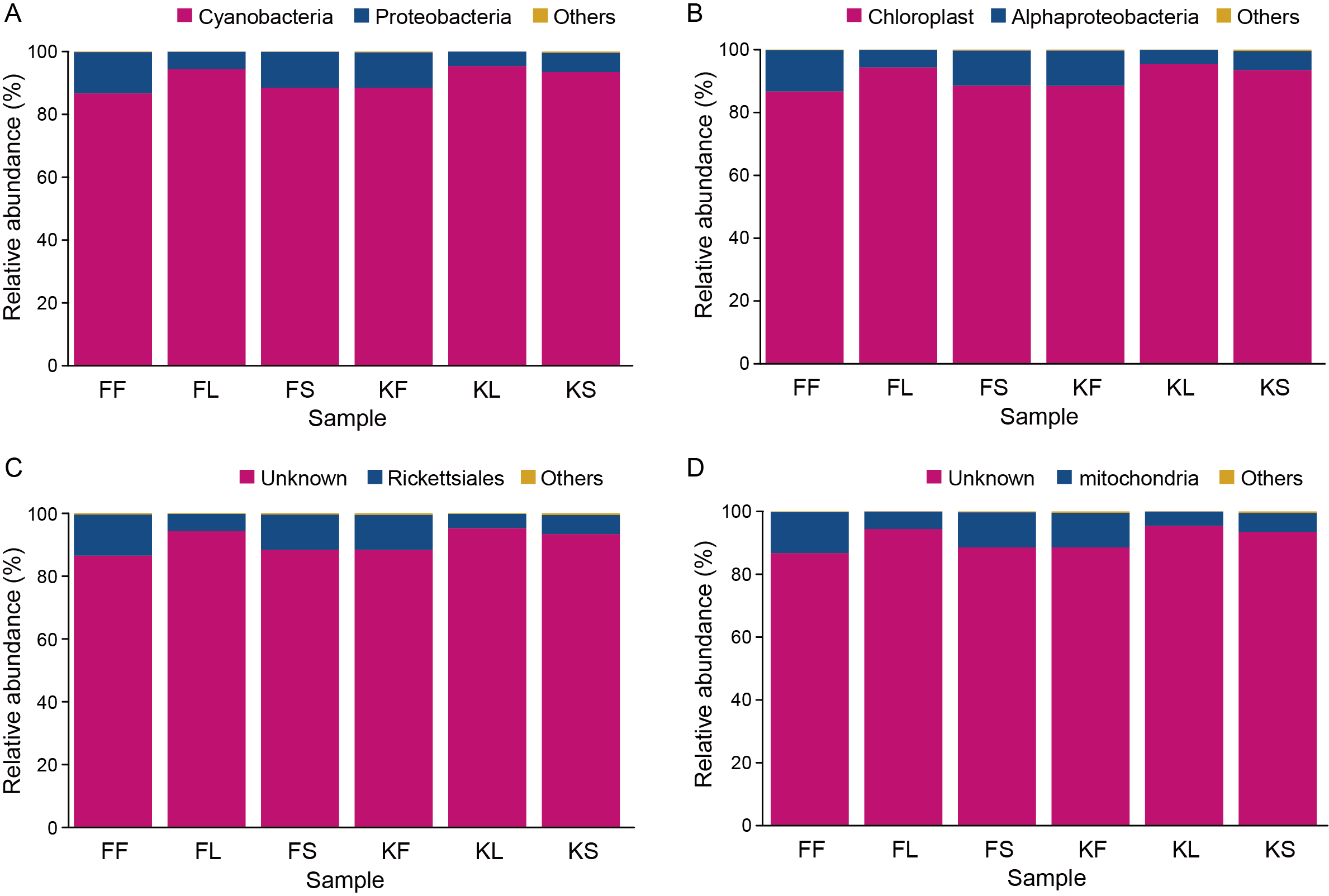

Supplement: S1 File — S1 Table. Statistics of sequencing data. S2 Table. Sequences of each representative OTU. S1 Fig. The length distribution of obtained clean tags in each sample (A: KF; B: FF; C: KL; D: FL; E: KS; F: FS). S2 Fig. Distribution of microorganism species at all levels of phylum (A), class (B), order (C), and family (D). S3 Fig. The Rarefaction curves (A) and the Shannon curves (B) of each sample. S4 Fig. Analysis of COG metabolic pathways in leaves between ‘Fengzao’ (FL) and ‘Kyoho’ (KL). S5 Fig. Analysis of COG metabolic pathways in stems between ‘Fengzao’ (FS) and ‘Kyoho’ (KS). (ZIP) [file pone.0290853.s001.zip › Supporting information/S2 Fig.tif]

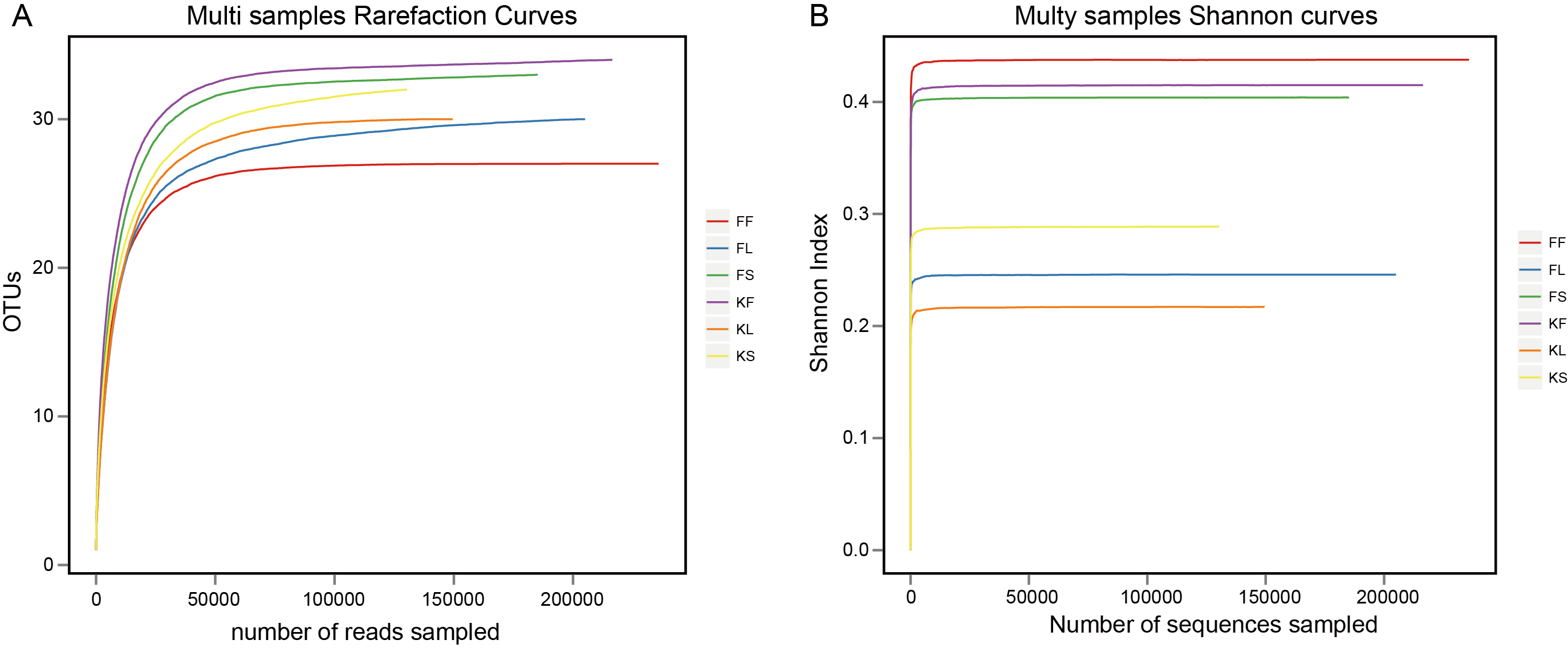

Supplement: S1 File — S1 Table. Statistics of sequencing data. S2 Table. Sequences of each representative OTU. S1 Fig. The length distribution of obtained clean tags in each sample (A: KF; B: FF; C: KL; D: FL; E: KS; F: FS). S2 Fig. Distribution of microorganism species at all levels of phylum (A), class (B), order (C), and family (D). S3 Fig. The Rarefaction curves (A) and the Shannon curves (B) of each sample. S4 Fig. Analysis of COG metabolic pathways in leaves between ‘Fengzao’ (FL) and ‘Kyoho’ (KL). S5 Fig. Analysis of COG metabolic pathways in stems between ‘Fengzao’ (FS) and ‘Kyoho’ (KS). (ZIP) [file pone.0290853.s001.zip › Supporting information/S3 Fig.tif]

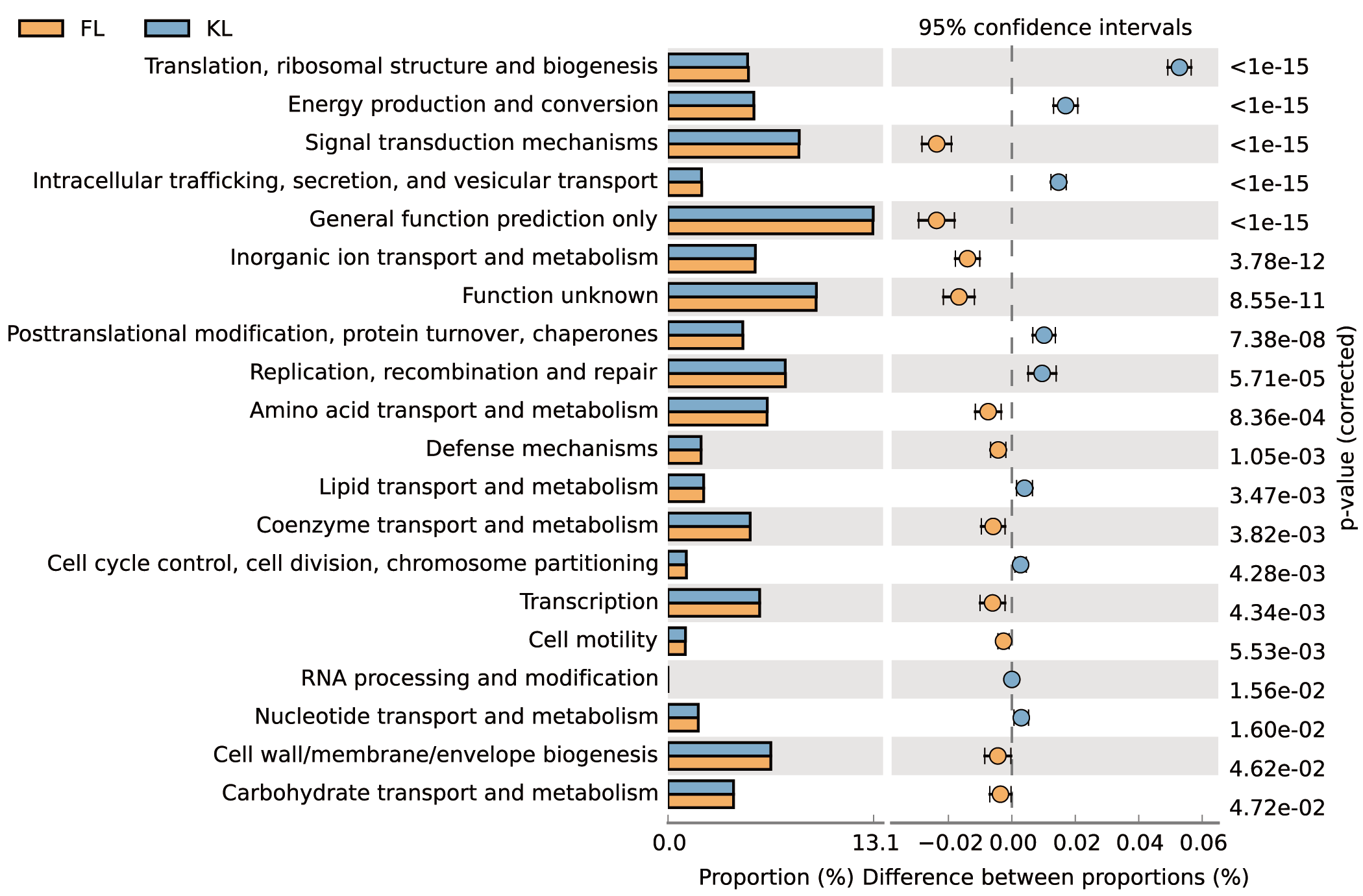

Supplement: S1 File — S1 Table. Statistics of sequencing data. S2 Table. Sequences of each representative OTU. S1 Fig. The length distribution of obtained clean tags in each sample (A: KF; B: FF; C: KL; D: FL; E: KS; F: FS). S2 Fig. Distribution of microorganism species at all levels of phylum (A), class (B), order (C), and family (D). S3 Fig. The Rarefaction curves (A) and the Shannon curves (B) of each sample. S4 Fig. Analysis of COG metabolic pathways in leaves between ‘Fengzao’ (FL) and ‘Kyoho’ (KL). S5 Fig. Analysis of COG metabolic pathways in stems between ‘Fengzao’ (FS) and ‘Kyoho’ (KS). (ZIP) [file pone.0290853.s001.zip › Supporting information/S4 Fig.tif]

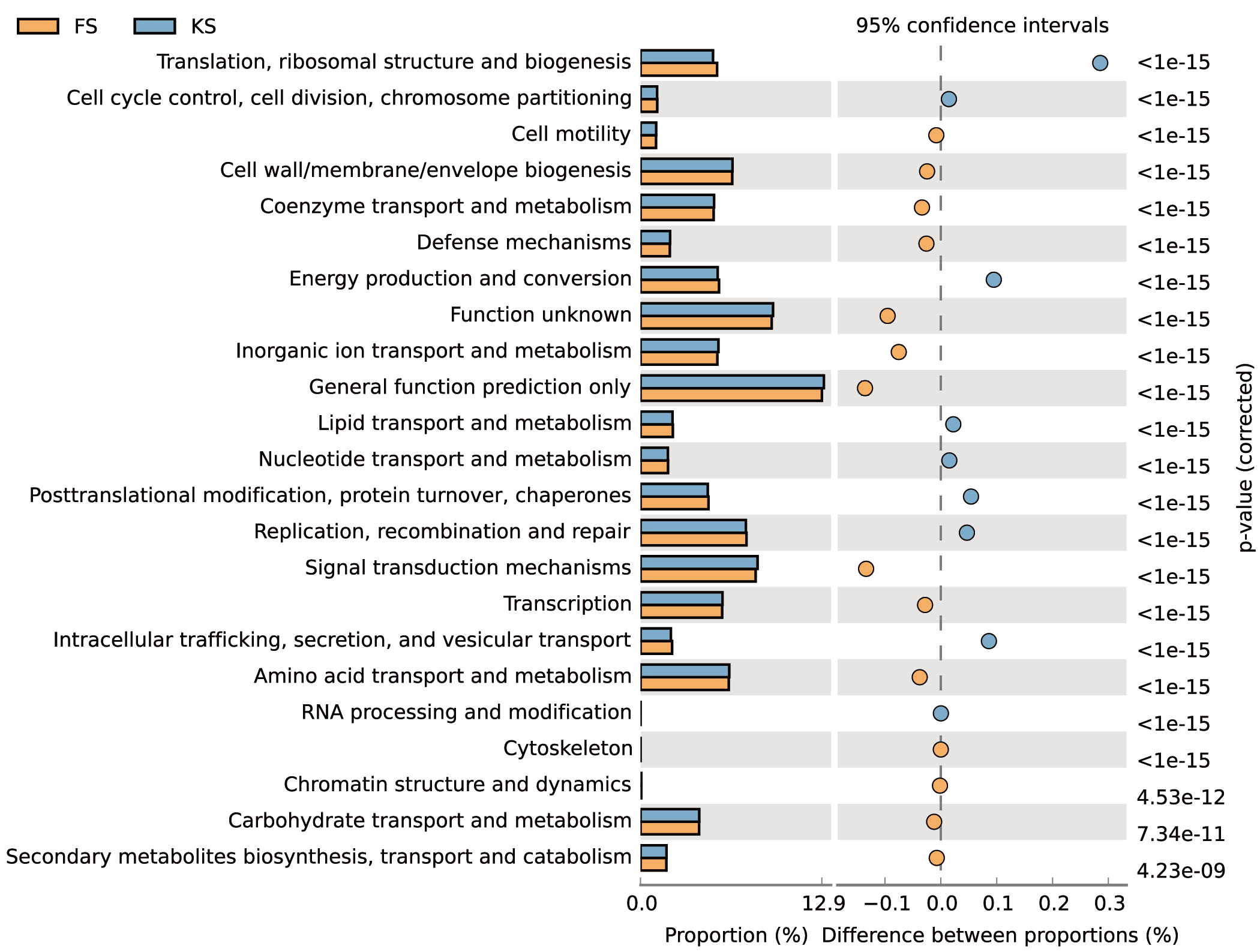

Supplement: S1 File — S1 Table. Statistics of sequencing data. S2 Table. Sequences of each representative OTU. S1 Fig. The length distribution of obtained clean tags in each sample (A: KF; B: FF; C: KL; D: FL; E: KS; F: FS). S2 Fig. Distribution of microorganism species at all levels of phylum (A), class (B), order (C), and family (D). S3 Fig. The Rarefaction curves (A) and the Shannon curves (B) of each sample. S4 Fig. Analysis of COG metabolic pathways in leaves between ‘Fengzao’ (FL) and ‘Kyoho’ (KL). S5 Fig. Analysis of COG metabolic pathways in stems between ‘Fengzao’ (FS) and ‘Kyoho’ (KS). (ZIP) [file pone.0290853.s001.zip › Supporting information/S5 Fig.tif]
